# Effect of acute ozone exposure on the lung metabolomes of obese and lean mice

**DOI:** 10.1371/journal.pone.0181017

**Published:** 2017-07-13

**Authors:** Joel Andrew Mathews, David Itiro Kasahara, Youngji Cho, Lauren Nicole Bell, Philip Ross Gunst, Edward D. Karoly, Stephanie Ann Shore

**Affiliations:** 1 Department of Environmental Health, Harvard T.H. Chan School of Public Health, Boston, Massachusetts, United States of America; 2 Metabolon Incorporated, Research Triangle Park, North Carolina, United States of America; National Research Council of Italy, ITALY

## Abstract

Pulmonary responses to the air pollutant, ozone, are increased in obesity. Both obesity and ozone cause changes in systemic metabolism. Consequently, we examined the impact of ozone on the lung metabolomes of obese and lean mice. Lean wildtype and obese *db/db* mice were exposed to acute ozone (2 ppm for 3 h) or air. 24 hours later, the lungs were excised, flushed with PBS to remove blood and analyzed via liquid-chromatography or gas-chromatography coupled to mass spectrometry for metabolites. Both obesity and ozone caused changes in the lung metabolome. Of 321 compounds identified, 101 were significantly impacted by obesity in air-exposed mice. These included biochemicals related to carbohydrate and lipid metabolism, which were each increased in lungs of obese versus lean mice. These metabolite changes may be of functional importance given the signaling capacity of these moieties. Ozone differentially affected the lung metabolome in obese versus lean mice. For example, almost all phosphocholine-containing lysolipids were significantly reduced in lean mice, but this effect was attenuated in obese mice. Glutathione metabolism was also differentially affected by ozone in obese and lean mice. Finally, the lung metabolome indicated a role for the microbiome in the effects of both obesity and ozone: all measured bacterial/mammalian co-metabolites were significantly affected by obesity and/or ozone. Thus, metabolic derangements in obesity appear to impact the response to ozone.

## Introduction

Ozone (O_3_) is a common air pollutant produced by exposure of automobile exhaust to sunlight. Exposure to O_3_ causes respiratory symptoms, increases susceptibility to pulmonary infections, and even increases the risk of mortality in those with underlying cardiorespiratory conditions [1–5]. O_3_ is a particular problem for asthmatics. Even O_3_ concentrations near the EPA standard are sufficient to reduce lung function in asthmatic children [1]. Hospital admissions and emergency room visits for asthma increase after days of high ambient O_3_ concentrations [6–8] and O_3_ causes airway hyperresponsiveness (AHR) [9], a canonical feature of asthma. O_3_ injures lung epithelial cells, and the ensuing inflammatory response, which includes production of numerous cytokines and chemokines and recruitment of neutrophils [10], likely also contributes to the capacity of O_3_ to trigger asthma.

Obesity amplifies the impact of O_3_ on the lungs. O_3_-induced increases in asthma symptoms are greater in obese than lean children [11]. Similarly, O_3_-induced reductions in lung function are greater in obese than lean adult human subjects, especially if those subjects also exhibit AHR [12,13]. Similar results are obtained in mice. Obese mice exhibit innate AHR [14]. O_3_-induced increases in airway responsiveness and in pulmonary neutrophil recruitment are also greater in obese than in lean mice [15–17]. The mechanistic basis for these augmented responses to O_3_ remains incompletely understood.

Metabolomic profiling offers a means of discovering metabolic pathways that underlie disease. For example, the importance of trimethylamine N-oxide (TMAO) for cardiovascular disease was first predicted from metabolomic profiling studies [18]. Obesity is a metabolic disease and there are marked effects of obesity on the serum and urinary metabolomes in humans, rats, and mice including changes in carbohydrate, lipid, and branched chain amino acid (BCAA) metabolism [19–21]. Lungs of naive obese mice also exhibit metabolic changes, including changes in lipid, phospholipid, and cholesterol metabolism [22]. In rats, acute exposure to O_3_ causes profound changes in the serum metabolome including increases in sugars, free fatty acids, BCAAs (valine, leucine, and isoleucine, and urea, indicating impaired glycemic control, lipolysis, and proteolysis [23]. Similar results are obtained in human subjects [24]. The lipid mobilization and increased glucose induced by O_3_ are consistent with effects of O_3_ on adipose tissue and liver, tissues that are substantially altered in obese mice. Indeed, transcriptomic profiling of livers from O_3_ exposed mice confirmed increased expression of genes involved in gluconeogenesis and decreased expression of genes involved in triglyceride biosynthesis [23]. Importantly, these systemic effects of O_3_ appear to contribute to O_3_-induced injury and inflammation within the lungs [25]. Nevertheless, effects of O_3_ on the *lung* metabolome have not been described in either lean or obese mice, though changes in the lung metabolome do accompany pulmonary exposures to other inhaled irritants and infectious agents [26–30].

The purpose of this study was to perform global metabolomic profiling on lungs of obese *db/db* mice and their lean wildtype (C57BL/6J) controls exposed to air or O_3_ in order to identify metabolites that could be contributing to the augmented responses to O_3_ observed in obese mice [15–17]. Mice were exposed to room air or to O_3_ (2 ppm) for 3 hours. Twenty-four hours later, the lungs were harvested, flushed with PBS to remove blood, flash frozen in liquid nitrogen, and analyzed via liquid-chromatography or gas-chromatography coupled to mass spectrometry for metabolites. The dose and timing of exposure were chosen to correspond with exposure conditions for which functional responses to O_3_ have already been established [16]. Our results indicate profound differences in the lung metabolomes of unexposed obese and lean mice that include elevations in lipids and carbohydrates. These changes may be due to elevations in these moieties in the blood [20] and subsequent diffusion into the lung extracellular fluid. O_3_ also affected the lung metabolome. Importantly, there were differential effects of O_3_ in obese and lean mice, including effects on BCAA metabolites, lysolipids, and glutathione. The lung metabolome also indicated a role for the microbiome in the effects of obesity on pulmonary responses to O_3_.

## Methods

### Mice

These studies were approved by the Harvard Medical Area Standing Committee on animals. Female *db/db* mice on a C57BL/6 background and age- and sex-matched wildtype (WT) C57BL/6J mice were purchased from The Jackson Labs at age 6 weeks, allowed to acclimatize within the mouse vivarium at the Harvard T.H. Chan School of Public Health for 4 weeks, and studied at 10 weeks of age. A separate cohort of female WT and *db/db* mice that were bred and raised in house were used for measurements of serum insulin. *Db/db* mice lack the longform of the receptor for the satiety hormone, leptin.

### Ozone exposure

For metabolomics, *db/db* and WT mice were exposed for 3 hours to room air or to O_3_ (2 ppm) in stainless steel and plexiglass exposure chambers [31]. During exposure, mice were placed within individual wire mesh cages and food and water were withdrawn. Mice were returned to their home cages immediately after exposure at which time food and water were restored. Mice were euthanized with an overdose of sodium pentobarbital 24 hours after cessation of exposure.

### Tissue harvest and processing

For mice in the metabolomics study, after euthanasia, blood was obtained by cardiac puncture for the preparation of serum. The chest wall was then opened and a small incision was made in the left ventricle. 10 ml of ice cold PBS was gradually injected into the right ventricle in order to flush blood from the lungs. The lungs were then flash frozen in liquid nitrogen and stored at -80°C until shipped on dry ice to Metabolon Inc. (Durham, NC). Upon receipt, the lungs were again frozen at -80°C until analysis.

In another cohort, *db/db* and WT mice were exposed to room air or O_3_ as described above. Blood was obtained and the lungs flushed of blood and frozen in liquid nitrogen as described above. In this cohort, lung tissue was used to prepare RNA for microarray and qRT-PCR analysis. Microarray data have been deposited at http://www.ncbi.nlm.nih.gov/geo/query/acc.cgi?acc=GSE81800).

### Metabolomics

The automated MicroLab STAR^®^ system from Hamilton Company was used to prepare samples for metabolomics profiling. Equal weight of lungs was used from each mouse. Prior to the first step in the extraction process, recovery standards were added for quality control (QC) purposes. A series of organic and aqueous extractions proprietary to Metabolon Inc. was then used to remove protein while allowing maximum recovery of small molecules. Extracts were divided into two fractions. One was used for analysis by liquid chromatography (LC) and the other was used for analysis by gas chromatography (GC). Samples were placed briefly on a TurboVap^®^ (Zymark) to remove the organic solvent, frozen, and dried under vacuum. Samples were then prepared for the appropriate instrument, either LC/MS (mass spectrometry) or GC/MS, as described by Evans et al [32]. For a brief description of the methods used for LC/MS and GC/MS, see the S1 Method. After analysis, raw data were extracted, peak-identified and QC processed using Metabolon's proprietary hardware and software. At the time of this analysis, identification of known chemical entities was based on comparison to metabolomic libraries of more than 1000 commercially available purified standard compounds. The combination of chromatographic properties and mass spectra gave an indication of a match to the specific compound or an isobaric entity. This data is available at the NIH Common Fund's Metabolomics Data Repository and Coordinating Center (supported by NIH grant, U01-DK097430) website, the Metabolomics Workbench, http://www.metabolomicsworkbench.org ID:934.

### RNA extraction and real time PCR

After excision, the right lung was immersed in RNAlater (Qiagen) for subsequent preparation of RNA [33]. A small volume spectrophotometer (Nanodrop, Thermo Scientific) was used to assess RNA concentration and purity and a commercial kit (SuperScript III for qRT-PCR, Invitrogen) was used to convert RNA into cDNA. *Gclc* mRNA abundance was quantified using real time PCR (7300 Real-Time PCR Systems, Applied Biosystems) with SYBR-green detection and normalized to *36B4* ribosomal RNA (*Rplp0)*. Primers for *Gclc* were forward–TGTGGTATTCGTGGTACTGCT and CTGGGCCACTTTCATGTTCTC. Primers for *Gsta1* were forward: ACCTGATGCACTCCATTCTG and reverse: GCTGGACTGTGAGCTGAGTG. Primers for *Rplp0* were as described [33]. The ΔΔCt method was used to assess changes in mRNA abundances.

### ELISA

Serum was analyzed for insulin using an ELISA assays (EMD Millipore) according to the manufacturers’ recommendations.

### Statistics

For mRNA abundances and serum analytes: factorial ANOVA using genotype and expression as main effects and Fisher’s LSD test as follow up, was used to assess the significance of differences. For these outcomes, p<0.05 was considered statistically significant.

For metabolomics data analysis: missing values when present due to being under the limit of detection of the instruments were imputed with the minimum value on a per metabolite basis. For each metabolite, raw peak area counts were rescaled to set the median across all samples for that metabolite to 1 and the data log transformed. Then, a two way ANOVA consisting of the factors genotype and ozone treatment. Follow-up pairwise contrasts were also conducted to compare individual group means using F-tests. Storey’s q-values were calculated to estimate the proportion of false positives (see S1 Table for p and q values for each metabolite). PCA analysis was performed using the prcomp function in R 3.4 [34] (https://www.R-project.org/) and plotted with gglot2 package [35].

For metabolic pathway enrichment analysis, enrichment factors (EF) were calculated as follows where the significance of individual metabolites was assessed as p<0.05:

$$\text{EF = }\frac{\#\text{ of significantly affected metabolites in pathway / total }\#\text{ of metabolites in pathway}}{\text{total }\#\text{ of significantly affected metabolites / total }\#\text{ of detected metabolites}}$$

Fisher’s exact test was used to assess the significance of pathway or superpathway enrichment. A false discovery rate (FDR) was computed to account for multiple comparisons.

A p-value or q value <0.05 was considered statistically significant depending on whether individual metabolites (q value) or metabolites within a significantly affected pathway (per FDR analysis) (p value) were being assessed.

## Results

**Fig 1:**
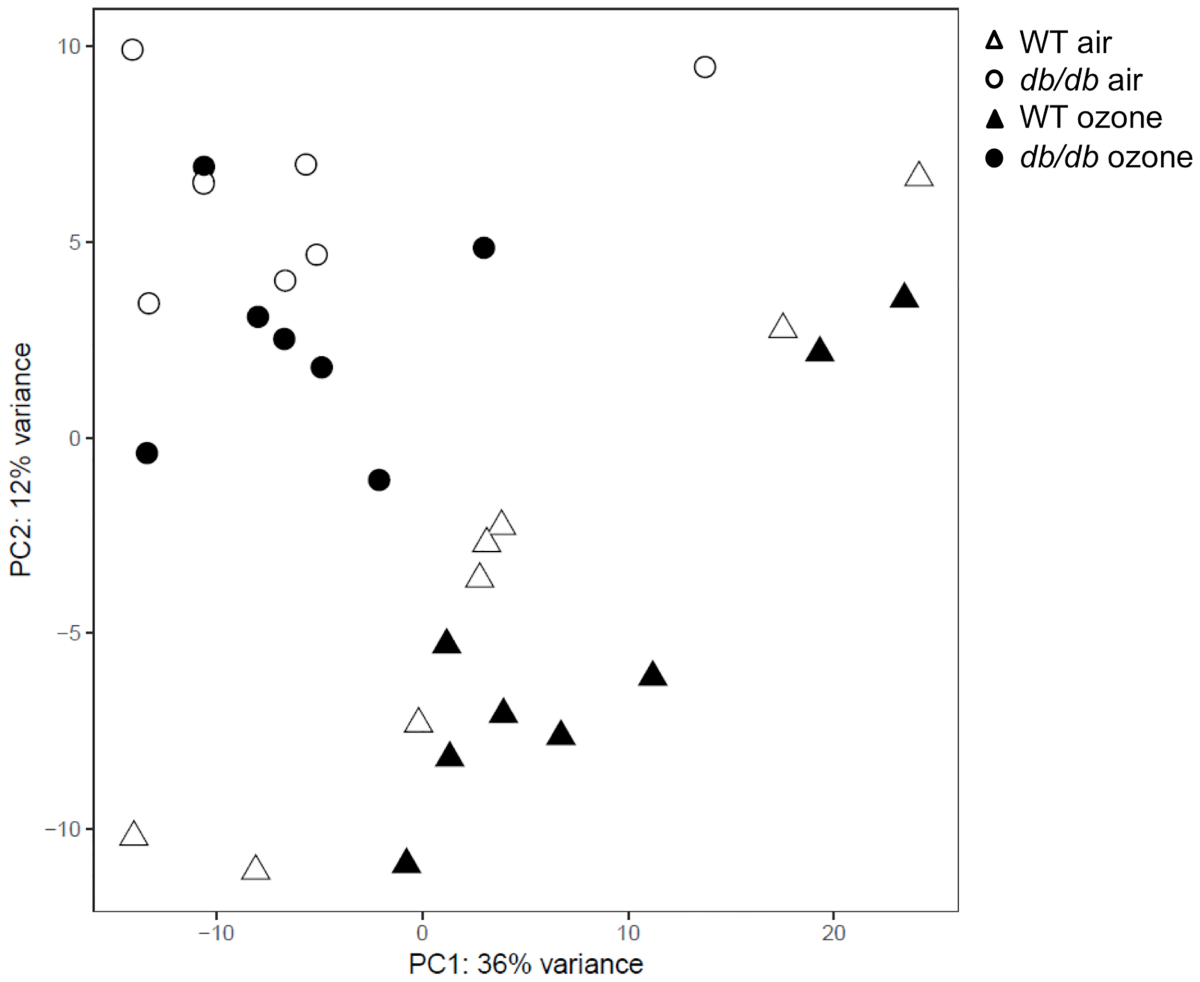
Principal component analysis of lung metabolites from lean wildtype (WT) and obese *db/db* mice exposed to air or ozone (2 ppm for 3 h) and studied 24 h after exposure.

Among the 321 biochemicals identified in lung tissue, two-way ANOVA identified 171 that were significantly affected by db genotype, 71 that were affected by O_3_ exposure, and 14 for which there was an interaction between genotype and O_3_. Differences between individual experimental groups are shown in Table 1. Both in air and O_3_ exposed mice, approximately 1/3 of identified biochemicals were different in *db/db* versus WT mice, with the majority being increased in the *db/db* mice. Principal component analysis (PCA) indicated a clear separation in the lung metabolomes of the *db/db* versus WT mice, both when all 4 groups were considered together (Fig 1), and when either the air-exposed or O_3_-exposed mice were considered separately (S1 Fig). O_3_ exposure also caused significant changes in lung biochemicals in both WT and *db/db* mice, with the majority being reduced in the O_3_- versus air-exposed mice (Table 1).

**Table 1:** Total number of lung tissue biochemicals (among 321 identified) that were significantly affected (*p*<0.05) by obesity and ozone.

	*db/db* WT		Ozone Room air	
	Room air	Ozone	WT	*db/db*
Total biochemicals *p*≤0.05	101	128	75	45
Biochemicals (↑/↓)	86|15	116|12	21|54	17|28

Below, we first describe the metabolic pathways affected by db genotype and by O_3_ exposure using metabolic pathway enrichment analysis of the significantly affected metabolites from the two-way ANOVA (Table 2). We then describe the results of a similar analysis of metabolic pathway enrichment using the results of the analysis comparing the 4 individual groups (Table 3). Next we discuss the lung metabolomes of air-exposed *db/db* versus WT mice, the lung metabolomes of WT mice exposed to air versus O_3_, and the differential impact of O_3_ exposure on the lung metabolomes of *db/db* versus WT mice, in each case focusing on those metabolites in pathways identified in Tables 2 and 3. Lastly, we describe the impact of obesity and O_3_ on metabolic substrates used for energy production, metabolites related to oxidative stress, and on bacterial mammalian co-metabolites, since these categories include metabolites from numerous different metabolic pathways.

**Table 2:** Metabolic pathways affected by db genotype and by O_3_ exposure.

Superpathway	Pathway	n	Genotype effect			Exposure effect		
			EF	p	FDR	EF	p	FDR
Amino acids and peptides		85	0.93	0.24	0.33	1.12	0.30	0.37
	Valine, leucine, and isoleucine metabolism	9	**1.88**	**0.0039**	**0.025**	1.51	0.33	0.39
Carbohydrates		34	**1.38**	**0.0092**	**0.034**	0.66	0.19	0.29
	Fructose, mannose, galactose, starch, and sucrose	9	**1.88**	**0.0031**	**0.025**	0.57	0.37	0.42
	Glycolysis, gluconeogenesis, pyruvate metabolism	9	**1.88**	**0.0031**	**0.025**	0	0.102	0.26
Energy		6	0.94	0.59	0.59	0	0.23	0.33
Lipids		137	0.99	0.46	0.50	**1.32**	**0.0064**	**0.033**
	Fatty acids	38	1.28	0.033	0.11	0.59	0.11	0.26
	Lysolipids	38	1.18	0.15	0.29	**2.38**	**0.00009**	**0.0023**
	Carnitine metabolism	7	0.54	0.18	0.29	**3.22**	**0.0084**	**0.034**
Nucleotides		28	0.74	0.088	0.25	1.45	0.17	0.29
Cofactors and vitamins		21	1.16	0.28	0.36	1.51	0.16	0.29
Xenobiotics		10	0.94	0.54	0.56	1.81	0.16	0.29

n: number of metabolites in pathway; Enrichment factor (EF) was computed as follows: (# of significant metabolites (by p value) in pathway/ total # of detected metabolites in pathway)/ (total # of significant metabolites/total # of detected metabolites); p values indicate the significance of enrichment of the metabolite group compared to the total number of significantly affected metabolites and were computed by Fisher’s exact test; FDR: false discovery rate; significantly affected pathways (FDR<0.1) are highlighted in bold text

**Table 3:** Metabolites enriched in wildtype and *db/db* mice exposed to air or ozone (O_3_).

Super pathway	Pathway	n	*Db/db* vs WT Air			*Db/db* vs WT Ozone			Ozone vs Air WT			Ozone vs air *Db/db*		
			EF	p	FDR	EF	p	FDR	EF	p	FDR	EF	p	FDR
Amino acids and peptides		85	0.64	0.008	.083	0.89	0.19	0.32	0.81	0.16	0.29	0.84	0.11	0.24
	Valine, leucine, and isoleucine metabolism	9	0.71	0.43	0.51	1.95	0.027	0.12	1.90	0.14	0.27	0	0.26	0.40
Carbohydrates		34	1.68	0.021	0.12	1.40	0.035	0.13	0.50	0.064	0.18	0.42	0.46	0.51
	Fructose, mannose, galactose, starch, and sucrose	9	**2.47**	**0.0052**	**0.070**	**2.23**	**0.0033**	**0.070**	0.95	0.65	0.65	0.79	0.64	0.65
	Glycolysis, gluconeogenesis, pyruvate metabolism	9	2.12	0.030	0.12	1.95	0.023	0.12	0	0.095	0.22	0	0.26	0.4
Energy		6	0.53	0.30	0.42	1.25	0.45	0.51	0	0.2	0.33	0	0.4	0.51
Lipids		137	1.21	0.021	0.12	0.99	0.49	0.52	1.19	0.072	0.19	1.04	0.46	0.51
	Fatty acids	38	**2.01**	**0.0002**	**0.010**	1.25	0.12	0.25	0.45	0.048	0.15	0.38	0.097	0.22
	Lysolipids	38	0.60	0.04	0.13	1.40	0.031	0.12	**1.91**	**0.0054**	**0.070**	1.50	0.18	0.31
Nucleotides		28	0.68	0.16	0.29	0.63	0.07	0.19	1.07	0.49	0.52	1.78	0.08	0.2
Cofactors and vitamins		21	0.76	0.30	0.42	1.19	0.30	0.42	0.85	0.43	0.51	0.70	0.41	0.51
Xenobiotics		10	0.64	0.34	0.47	1.25	0.362	0.47	2.57	0.013	0.11	3.06	0.04	0.13

n: number of metabolites in pathway; Enrichment Factor (EF) was computed as follows: (# of significant metabolites (by p value) in pathway/ total # of detected metabolites in pathway)/ (total # of significant metabolites/total # of detected metabolites); BCAA; branched chain amino acids; p values indicate the significance of enrichment of the metabolite group compared to the total number of significantly affected metabolites and were computed by Fisher’s exact test; FDR: false discovery rate; significantly enriched pathways (using a FDR<0.10) are in bold text

### Metabolic pathways affected by db genotype and by O_3_ exposure

Lung metabolites significantly affected by db genotype or by O_3_ exposure in the two-way ANOVA are highlighted in blue in S1 Table. The metabolic superpathways and pathways to which these metabolites belong are also indicated in S1 Table. In order to determine whether there were metabolic pathways that were enriched among those metabolites affected by db genotype or by O_3_, we calculated an enrichment factor (Table 2) for each superpathway, and for affected pathways that were part of any significantly affected superpathway or for which there were pre-existing data to suggest an effect of O_3_ or obesity (e.g. metabolism of BCAAs). Enrichment factors were calculated as described in the methods. Pathways with a significant enrichment of significantly altered metabolites (as indicated by an FDR < 0.1) are indicated in bold text in Table 2. This analysis indicated a significant effect of db genotype on carbohydrate metabolism, particularly metabolites involved in fructose, mannose, galactose, starch, and sucrose metabolism and metabolites involved in glycolysis, gluconeogenesis, and pyruvate metabolism. Metabolites involved in BCAA metabolism were also affected by db genotype. Enrichment factor analysis also indicated a significant effect of O_3_ exposure on lipid metabolites, particularly lysolipids and metabolites involved in carnitine metabolism.

We performed a similar analysis on lung metabolites identified as being significantly affected by db genotype in either air or O_3_ exposed mice or significantly affected by O_3_ exposure in either WT or *db/db* mice (Table 3). These metabolites are highlighted in red (increased) and green (decreased) in S1 Table. Enrichment factor analysis indicated that the effect of db genotype on carbohydrate metabolism was observed in both air and O_3_ exposed mice and that there was also an effect of db genotype on fatty acids in the air but not O_3_ exposed mice. The data also indicated that the effect of O_3_ exposure on lysolipids was limited to the WT mice.

The specific metabolites in the pathways identified in Tables 2 and 3 are discussed in greater detail below.

### Differences in the lung metabolomes of db/db and WT mice during room air exposure

**Fig 2:**
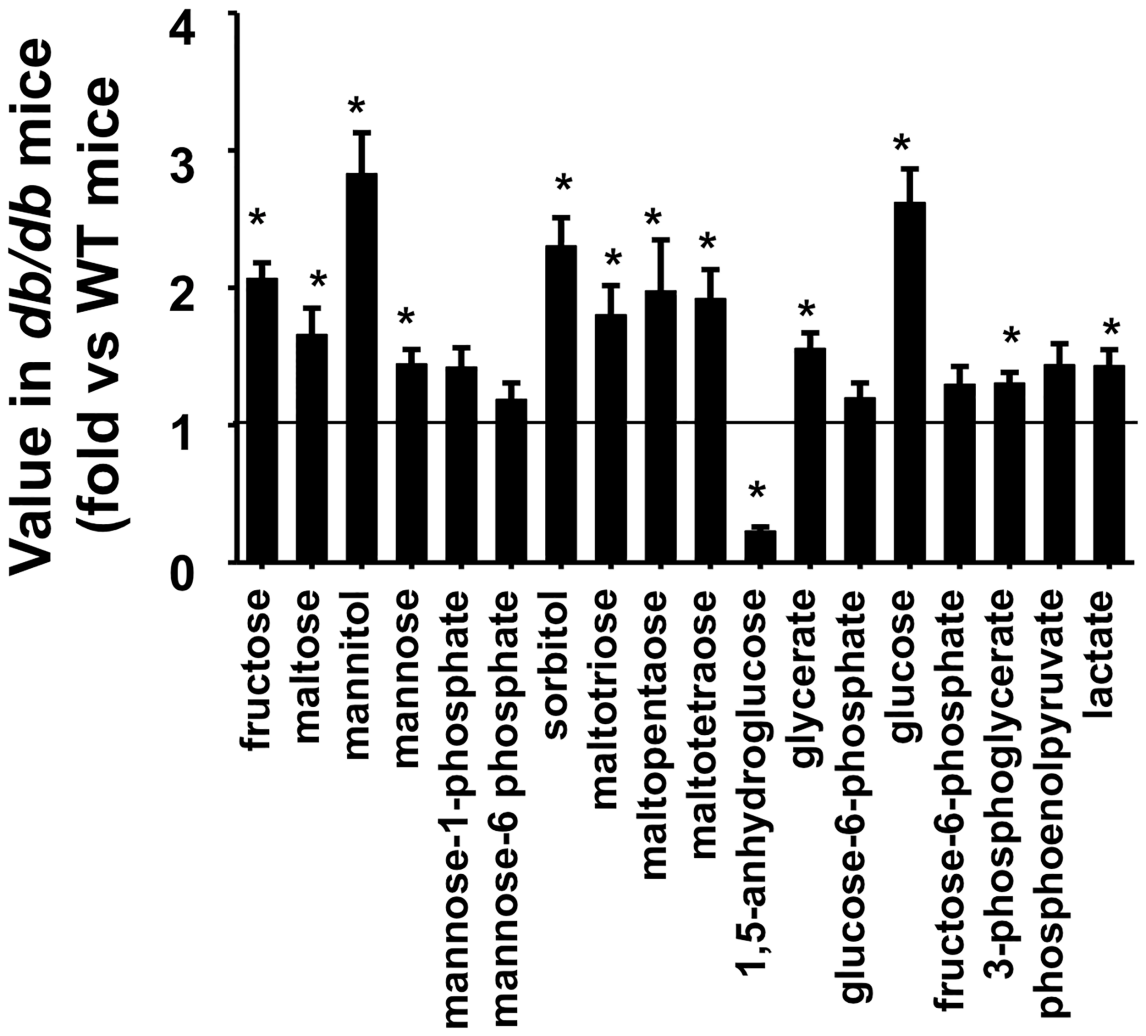
Lung carbohydrates in *db/db* mice exposed to air. Data are expressed relative to the mean values in WT mice. Results are mean ± SE of data from 8 mice/group. * p<0.05 versus air exposed WT mice.

As described above, pathway enrichment analysis indicated that metabolites related to carbohydrate and lipid metabolism were different in lungs of *db/db* and WT mice exposed to room air (Table 3). This metabolomic profile is similar to that reported in the blood of *db/db* versus WT mice [20]. The carbohydrates involved were primarily those related to glucose metabolism and the metabolism of other simple sugars (Table 3 and S1 Table). As shown in Fig 2, most of these metabolites were increased in the lungs of *db/db* versus WT mice, consistent with the systemic hyperglycemia and insulin resistance of *db/db* mice. Only 1,5-anhydroglucitol (1,5-AG) was reduced in the *db/db* mice (Fig 2), likely as a result of increased loss in the urine because of the high filtered glucose load in the kidney[20].

**Fig 3:**
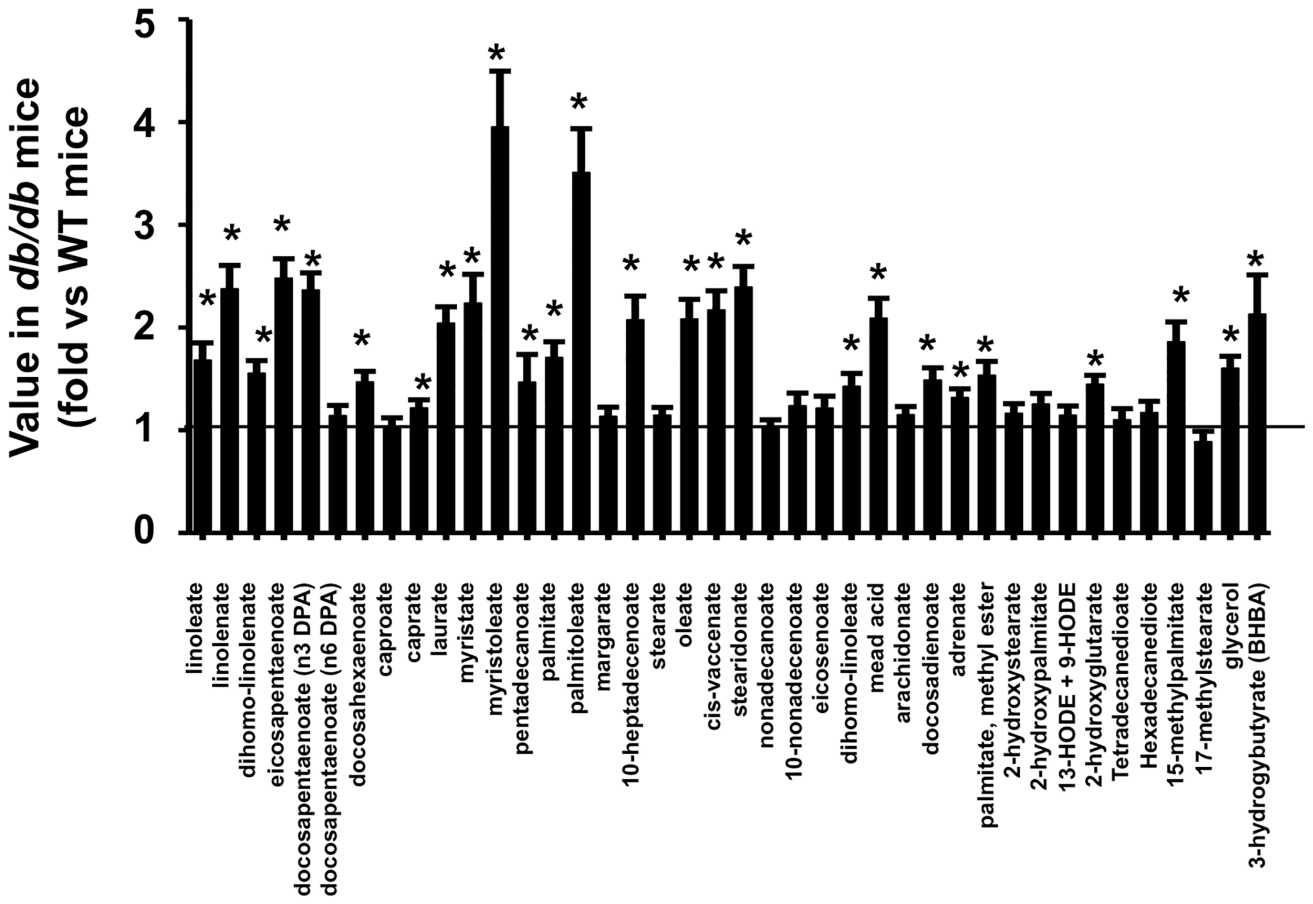
Lung fatty acids, glycerol, and ketones (BHBA) in *db/db* mice exposed to air. Data are expressed relative to the mean values in WT mice. Results are mean ± SE of data from 8 mice/group. * p<0.05 versus air exposed WT mice.

Lipids, particularly fatty acids, were also altered in the lungs of *db/db* versus WT mice exposed to air (Table 3). Most fatty acids were increased in *db/db* versus WT mice (Fig 3), as were glycerol, and the ketone BHBA (Fig 3), consistent with the marked systemic insulin resistance and consequent lipolysis characteristic of *db/db* mice. There were also changes in other lipids in the lungs of *db/db* mice (S1 Table). For example, although cholesterol itself was unchanged, there were elevations in several cholesterol metabolites, including dihydrocholesterol, 7-α-hydroxcholesterol, 7-β-hydroxycholesterol, and 7-ketocholesterol (S1 Table). Milner et al [22] also observed significant changes in many lipid moieties in lungs of obese versus lean mice, although their analytic methods differed from ours.

Other metabolites that differed in lungs of air exposed *db/db* versus WT mice are found in S1 Table.

### The effect of O_3_ exposure on the lung metabolome of WT mice

In WT mice, examination of metabolic pathways affected by O_3_ exposure indicated an effect on lysophospholipids (Table 3). In particular, O_3_ exposure caused substantial decreases in lysolipids in WT mice (Table 4). Of the 38 measured lysolipids, 17 were significantly lower in O_3_- than air-exposed WT mice, Notably, of the 17 lysolipids reduced by O_3_ in lean mice, most were choline-containing lysolipids; the phospholipids that make up the majority of the phospholipids in lung surfactant [36]. There was also a significant effect of O_3_ on the monoglycerides 1-linoleoylglycerol and 2-linoleoylglycerol (S1 Table).

**Table 4:** Effect of ozone exposure on lung lysolipids in obese and lean mice.

Lysolipid	*Db/db*/WT Air	*Dd/db*/WT Ozone	Ozone/Air WT	Ozone/Air *Db/db*
1-palmitoylglycerophosphoethanolamine	1.62	1.91*	0.97	1.14
2-palmitoylglycerophosphoethanolamine	1.84*	2.18*	0.90	1.07
1-stearoylglycerophosphoethanolamine	1.75	2.36	0.87	1.17
1-oleoylglycerophosphoethanolamine	1.96	2.72*	0.83	1.15
2-oleoylglycerophosphoethanolamine	1.97*	2.15*	0.92	1.01
1-linoleoylglycerophosphoethanolamine	1.36	1.72*	0.82	1.04
2-linoleoylglycerophosphoethanolamine	1.13	1.52	0.81	1.09
1-arachidonoylglycerophosphoethanolamine	1.87*	1.99*	0.90	0.95
2-arachidonoylglycerophosphoethanolamine	0.91	2.28	0.32#	0.81
2-docosapentaenoylglycerophosphoethanolamine	1.42	4.56	0.25#	0.74
2-docosahexaenoylglycerophosphoethanolamine	1.05	2.30	0.35#	0.78
1-stearoylglycerophosphoglycerol	1.89*	1.42*	1.37	1.03
1-myristoylglycerophosphocholine	1.29	3.10*	0.22#	0.53#
2-myristoylglycerophosphocholine	1.24	1.84	0.28#	0.42#
1-palmitoylglycerophosphocholine	1.67	2.21	0.53	0.69
2-palmitoylglycerophosphocholine	1.78	1.88	0.47#	0.50#
1-palmitoleoylglycerophosphocholine	1.63	6.94*	0.23#	0.97
2-palmitoleoylglycerophosphocholine	1.56	2.79	0.30#	0.54#
1-heptadecanoylglycerophosphocholine	2.88	2.57	0.83	0.93
1-stearoylglycerophosphocholine	2.02	2.45	0.53	0.65
2-stearoylglycerophosphocholine	3.70*	3.06	0.66	0.54#
1-oleoylglycerophosphocholine	2.33	3.92	0.38	0.63
2-oleoylglycerophosphocholine	2.31	2.20	0.64	0.61#
1-linoleoylglycerophosphocholine	1.27	3.78*	0.22#	0.65
2-linoleoylglycerophosphocholine	0.85	3.63*	0.21#	0.91
1-arachidoylglycerophosphocholine	1.37	2.90*	0.28#	0.60#
1-arachidonoylglycerophosphocholine	1.42	4.59*	0.21#	0.63
2-arachidonoylglycerophosphocholine	0.98	4.12	0.20#	0.85
1-docosapentaenoylglycerophosphocholine	2.50*	2.45*	0.55#	0.54#
2-docosapentaenoylglycerophosphocholine	1.92	6.55*	0.21#	0.73
1-docosahexaenoylglycerophosphocholine	1.11	5.12	0.23#	1.07
2-docosahexaenoylglycerophosphocholine	1.21	3.59	0.26#	0.77
1-palmitoylglycerophosphoinositol	1.48	2.15*	0.68	1.00
1-stearoylglycerophosphoinositol	1.75	2.41*	0.74	1.02
1-oleoylglycerophosphoinositol	2.07*	2.38*	0.91	1.11
1-arachidonoylglycerophosphoinositol	1.45	1.68*	0.75	0.87
2-arachidonoylglycerophosphoinositol	1.45	2.60*	0.57	0.99
1-palmitoylplasmenylethanolamine	1.60	2.60*	0.71	1.15

Results are the ratio of mean lysolipid scaled peak area in *db/db* versus wildtype (WT) mice exposed to air or ozone or in ozone versus air exposed WT or *db/db* mice.

*p<0.05 versus WT;

^#^ p<0.05 versus air exposed mice of the same genotype. n = 8/group

Other biochemicals were also significantly affected by O_3_ in WT mice (see S1 Table). For example, O_3_ exposure caused an approximate 2-fold increase in lung citrulline in WT mice, and a similar effect in *db/db* mice. Arginine is converted to citrulline by nitric oxide synthase (NOS). Hence, the increase in citrulline following O_3_ exposure is consistent reports of increased NOS expression following O_3_ exposure in mice [37]. O_3_ also caused a marked decrease in the lung heme in WT mice. The decrease in lung heme was likely the result of increased expression of heme-oxygenase, which catalyzes heme degradation, and which is known to increase following O_3_ exposure [33].

### Obesity-related differences in the effect of O_3_ exposure on the lung metabolome

There were several notable differences in the metabolites affected by O_3_ in *db/db* versus WT mice (Tables 2 and 3). For example, whereas there was a significant enrichment of lysolipids among the metabolites affected by O_3_ exposure in WT mice, this was not the case in *db/db* mice (Table 3), although 8 of 38 lysolipids were significantly lower in O_3_- than air-exposed *db/db* mice (Table 4). However, even for these latter lysolipids, the magnitude of the reduction was not as great in *db/db* as in WT mice. It is noteworthy that of the 8 lysolipids reduced by O_3_ in obese mice, all were choline-containing lysolipids. Similarly, whereas monoglycerides were elevated by O_3_ in WT mice no such effect was observed in *db/db* mice (S1 Table).

Hypotaurine, an osmolyte, was significantly increased after O_3_ in *db/db* but not WT mice (S1 Table). Others have reported reductions in hypotaurine in blood of *db/db* versus WT mice [38], indicating that the increases in lung hypotaurine were unlikely to derive from systemic sources. Hypotaurine acts as an antioxidant within the mammalian reproductive tract [39] and it is conceivable that elevated levels in *db/db* mice exposed to O_3_ are acting to protect the lungs from O_3_-induced oxidative damage.

Several other metabolites were different in *db/db* versus WT mice exposed to O_3_ even though no similar difference or trend was observed in *db/db* versus WT mice exposed to air. For example, the prostanoids PGE1, PGE2, PGI2, and 6-keto prostaglandin F1α were each significantly greater in *db/db* than WT mice exposed to O_3_ but not air (S1 Table). Many of the other metabolites in this category were lysolipids (see above) or were bacterial mammalian co-metabolites. The latter are discussed in more detail below.

### Substrates used for energy production

**Fig 4:**
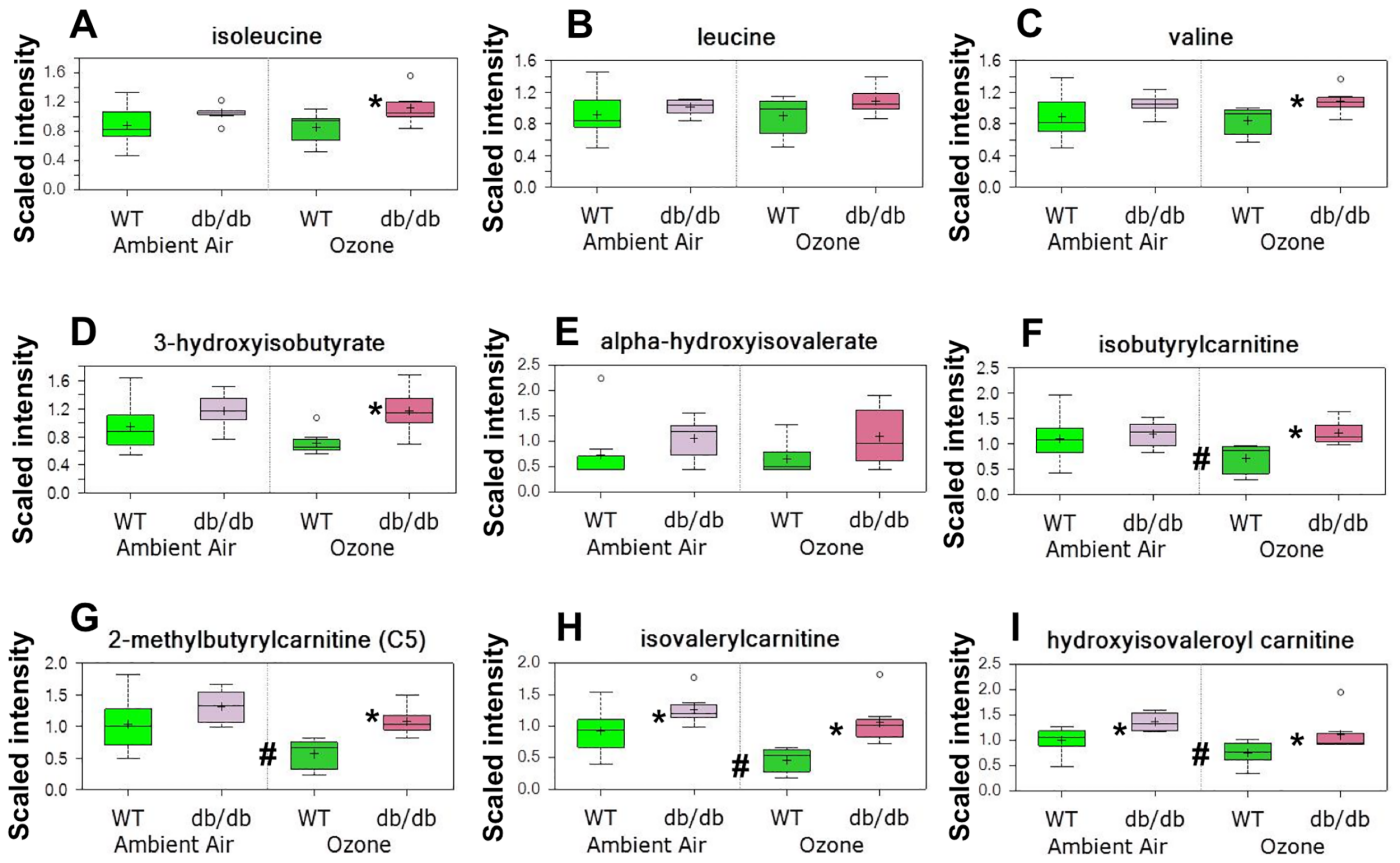
Lung branched chain amino acids and their metabolites in lungs of *db/db* and WT mice exposed to air and ozone. Results are presented as follows. The y axis is the scaled intensity calculated for each metabolite by taking the raw area counts rescaled so that the median across all mice was equal to 1. The + indicates the mean value and the line in the center of bar indicates the median value for each group. The upper and lower edges of the bar indicate the limits of the upper and lower quartile and the top and bottom of the error bars indicate the maximum and minimum of the distribution. Extreme data points are indicated by symbols outside of the maximum and minimum of the distribution. * p<0.05 versus exposure-matched WT mice. # p<0.05 versus genotype-matched air exposed mice.

Pathway enrichment analysis indicated a significant effect of db genotype on BCAA metabolism (Table 2). Two-way ANOVA indicated a significant effect of genotype on all 3 BCAAs (isoleucine, leucine, and valine) (Fig 4A, 4B and 4C), and the BCAA metabolites 3-hydroxyisobutyrate, alpha-hydroxyisovalerate, isobutyrylcarnitine, 2-methylbutyrylcarnitine, isovalerylcarnitine and hydoxyisovaleroylcarnitine (Fig 4D, 4E, 4F, 4G, 4H and 4I). Increases in BCAAs and their short chain acylcarnitine metabolites are typically observed in serum and tissues from obese mice and obese humans [19,40–42], and are thought to reflect reduced catabolism of BCAAs, a process that yields carbon sources that enter into energy production pathways. Indeed, BCAA metabolites were each higher in lungs of *db/db* versus WT mice (Fig 4). However, the effect was only observed after O_3_ exposure, in part because there were significant reductions in isobutyrylcarnitine, 2-methylbutyrylcarnitine, isovalerylcarnitine, and hydroxyisovaleroyl carnitine after O_3_ exposure in WT but not *db/db* mice (Fig 4F, 4G, 4H and 4I). Reductions in these BCAA metabolites are observed during catabolism of BCAAs [42]. Hence, these data suggest increased reliance of lungs of WT but not *db/db* mice on BCAA catabolism for energy production after O_3_ exposure.

**Fig 5:**
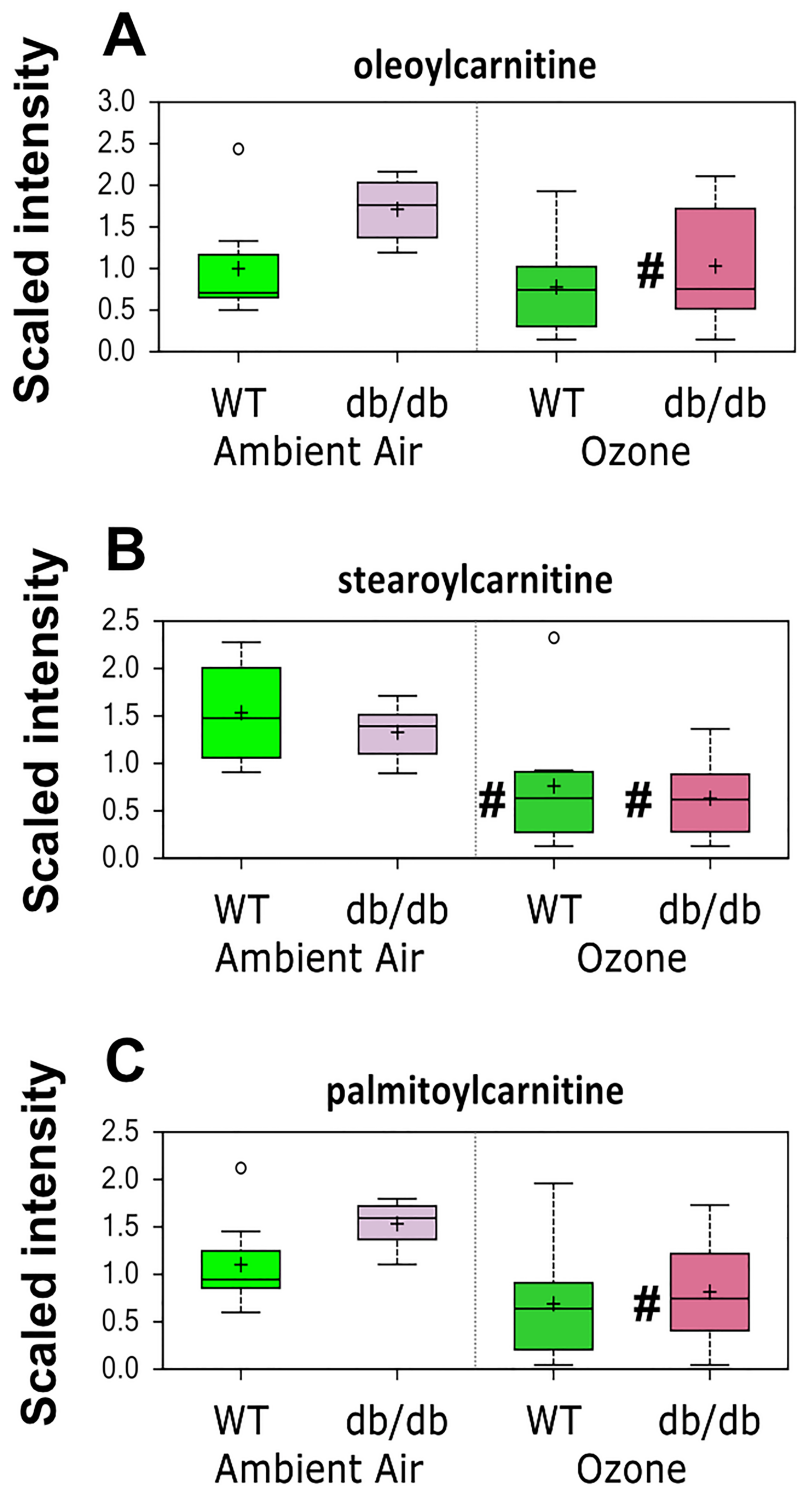
Lung long chain acylcarnitines in *db/db* and WT mice exposed to air or ozone. Results are expressed as described in Fig 4. n = 8/group * p<0.05 versus exposure-matched WT mice. # p<0.05 versus genotype-matched air exposed mice.

Pathway enrichment analysis also indicated a significant effect of O_3_ exposure on carnitine metabolism (Table 2). Conjugation of fatty acids with carnitine facilitates transport of these molecules across mitochondrial membranes where they can undergo subsequent β-oxidation. In particular, two-way ANOVA indicated that O_3_ caused a significant reduction in 3 of 3 measured long-chain acylcarnitines: oleoylcarnitine, stearoylcarnitine, and palmitoylcarnitine (Fig 5). The magnitude of the effect of O_3_ on these acylcarnitines was generally greater in the *db/db* than the WT mice: all 3 measured long-chain acylcarnitines were significantly reduced in obese mice after O_3_. However, a significant reduction in stearoylcarnitine was also reduced after O_3_ in lean mice (Fig 5A, 5B and 5C). Elevations in plasma acylcarnitines are typically observed when specific enzyme deficiencies prevent their metabolism via β-oxidation [43]. Consequently, reductions in oleoylcarnitine, stearoylcarnitine, and palmitoylcarnitine observed after O_3_ exposure likely reflect increased β-oxidation in lung cells.

**Fig 6:**
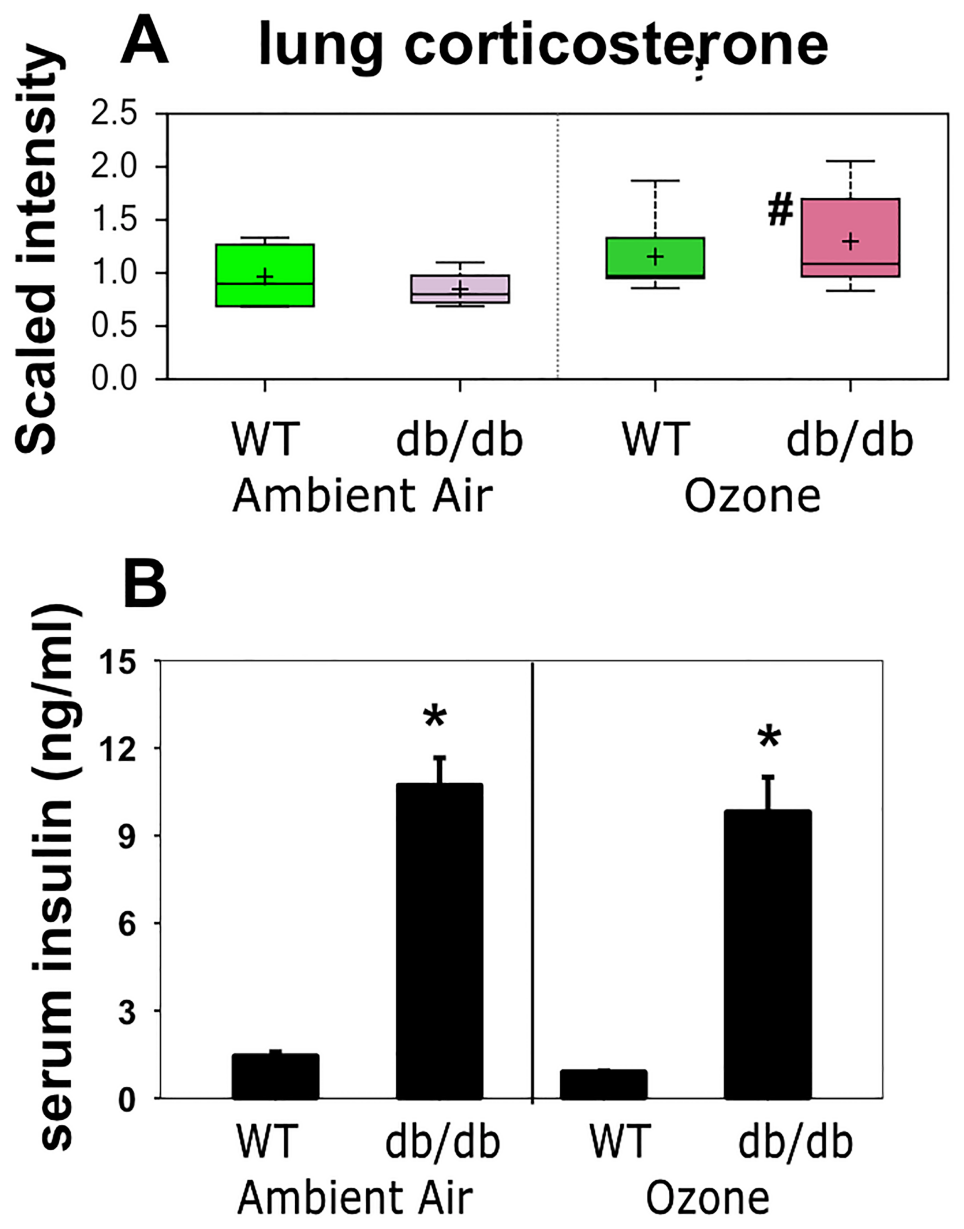
Lung corticosterone (A) and serum insulin (B) in *db/db* and WT mice exposed to air or ozone. For A, results are presented as described in Fig 4. For B, results are mean ± SE of data from 5–8 mice/group. * p<0.05 versus exposure-matched WT mice. # p<0.05 versus genotype-matched air exposed mice.

Metabolism regulating hormones: Since corticosteroids promote β-oxidation [44] and also attenuate BCAA catabolism [45], similar to the result obtained in obese O_3_ exposed mice (Figs 4 and 5), we hypothesized that greater O_3_-induced increases in corticosterone in *db/db* than WT mice might account for the different effects of O_3_ on β-oxidation (Fig 5) and BCAA metabolism (Fig 4) observed in *db/db* versus WT mice. Corticosterone was among the biochemicals identified in our metabolomic analysis and indeed two-way ANOVA did indicate greater lung corticosterone in O_3_- than air-exposed mice (Fig 6A and S1 Table), presumably as a result of increases in serum corticosterone. Notably, the effect of O_3_ only reached significance in the *db/db* mice.

Differences in insulin (Fig 6B) could also account for obesity-related differences in the effects of O_3_ on lung BCAA metabolism (Fig 4). *Db/db* mice are markedly resistant to insulin [20]. In human subjects, the development of insulin resistance is associated with increases in circulating BCAAs and reductions in their catabolism [19,41]. Thus, *db/db* mice should be limited in their ability to catabolize BCAAs, as observed (Fig 4). Consequently, we also measured serum insulin. Serum insulin was higher in *db/db* than WT, as expected, but there was no effect of O_3_ exposure on serum insulin in either group of mice (Fig 6B).

### Glutathione and other markers of oxidative stress

**Fig 7:**
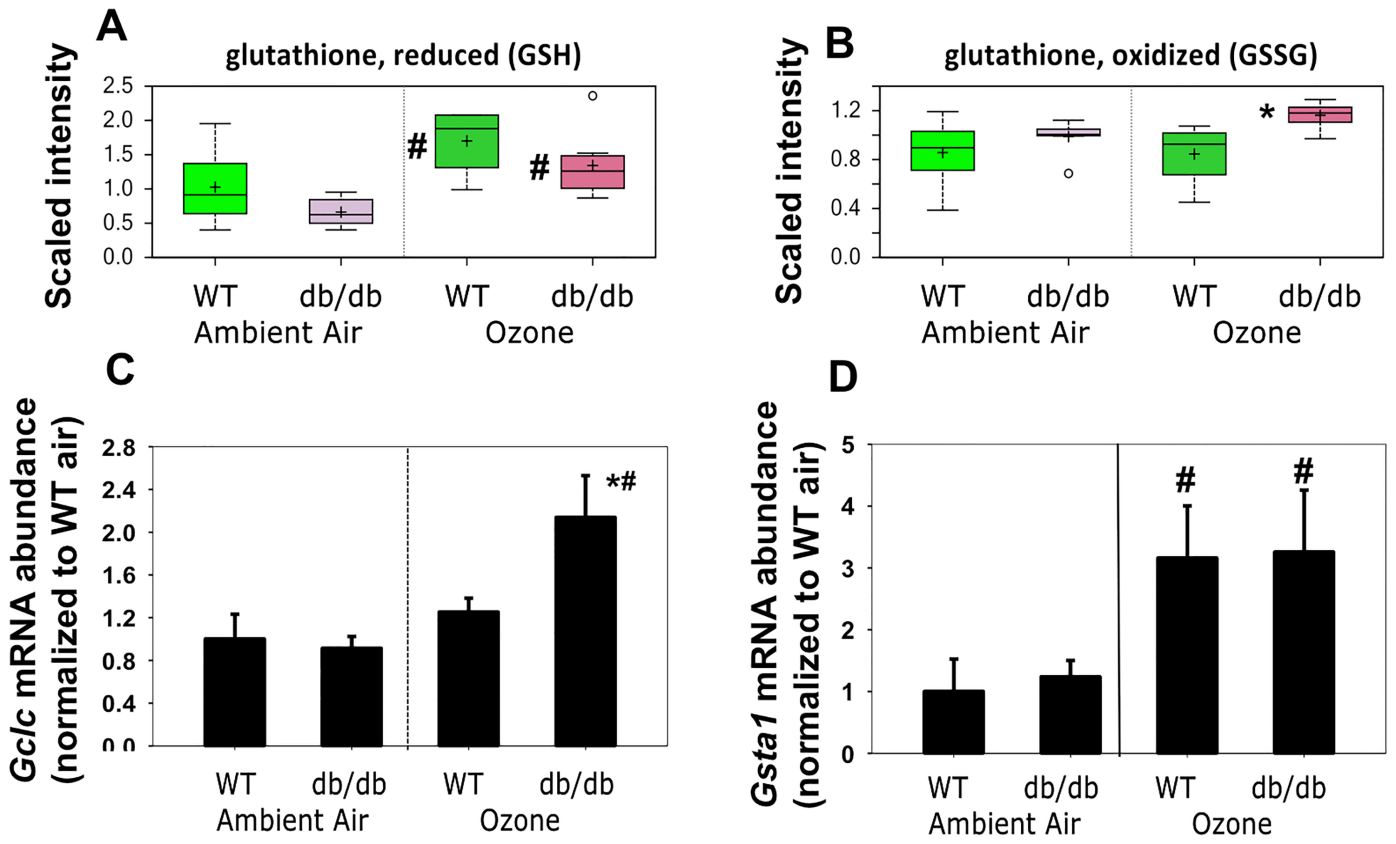
Lung GSH (A), GSSSG (B), as well as *Gclc* (C) and *Gsta1* (D) mRNA abundances in *db/db* and WT mice exposed to air or ozone. For A and B, data are presented as described in Fig 4, n = 8/group, # q<0.05 versus air; * q<0.05 versus WT. For C and D, results are mean ± SE, are presented relative to the WT air exposed values, and were obtained in lung tissue from a separate cohort of mice. n = 5-8/group. * p<0.05 versus exposure-matched WT mice. # p<0.05 versus genotype-matched air exposed mice.

O_3_ causes oxidative stress within the lungs, and an associated induction of anti-oxidant enzymes and systems [46]. There is also evidence of increased airway oxidative stress in obese asthmatics [47]. Consequently, we examined markers of oxidative stress among the lung metabolites identified. The tripeptide, glutathione (GSH), is a key component of the systems that maintain cell redox status and exposure to O_3_ caused changes in glutathione metabolism in lean and obese mice (Fig 7A and 7B). Compared to air, O_3_ caused a significant increase in GSH in lung tissue from both lean and obese mice (Fig 7A). GSH is produced by ligation of glutamate and cysteine by the catalytic subunit of glutamate—cysteine ligase (*Gclc*)(the rate limiting step) followed by addition of glycine by the enzyme glutathione synthetase (*Gss*). Survey of a microarray analysis we performed assessing lung gene expression in *db/db* mice exposed to air or O_3_ indicated no change in *Gss* mRNA expression in *db/db* mice after O_3_ (GSE81800). To determine whether increases in GSH were consequent to increased expression of *Gclc*, a redox-regulated gene [48], we measured pulmonary *Gclc* mRNA abundance by qRT-PCR. O_3_ did increase *Gclc* expression, but the effect was only observed in obese mice (Fig 7C). As the lung is one of the organs with the highest uptake of circulating GSH [49], it is possible that the increase in GSH after O_3_ (Fig 7A), especially in lean mice, is derived from non-pulmonary production of GSH rather than an increase in lung-derived GSH.

Given the observed increases in GSH with O_3_ (Fig 7A), we also examined O_3_-induced changes in pulmonary expression of glutathione-S-transferases (Gst), to determine whether there were likely to be obesity-related differences in the glutathionylation of targets such as lipid peroxides that are generated by O_3_ exposure. Using our microarray data, we identified two Gst genes that were highly expressed in the lungs and also significantly changed by O_3_: *Gsta1 and Gsta2*. RT-PCR confirmed increased expression of *Gsta1* after O_3_, but there was no difference in the impact of O_3_ on *Gsta1* in lean versus obese mice (Fig 7D).

Under conditions of oxidative stress, GSH is converted to oxidized GSH (GSSG)[48]. Although significant O_3_-induced increases in GSSG were not observed in either lean or obese mice, levels of GSSG were significantly higher in obese mice versus lean mice exposed to O_3_ (Fig 7B).

Others have proposed that airway oxidative stress in obese asthmatics may be the result of NOS uncoupling, which results in the production of superoxide anion instead of nitric oxide [50]. In particular, NOS uncoupling occurs when L-arginine is reduced or when asymmetric dimethyl arginine (ADMA) is increased. However, we did not observe any significant effect of either obesity or O_3_ on lung arginine or ADMA levels (S1 Table), though we cannot rule out the possibility that there were changes in these moieties in the blood.

**Fig 8:**
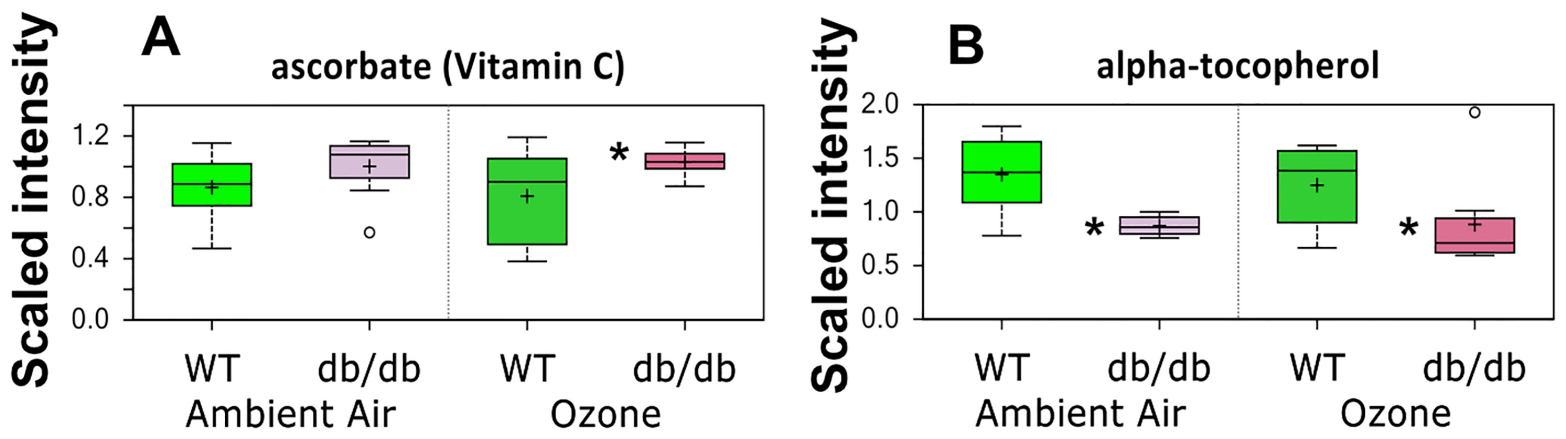
Lung ascorbate (A) and alpha-tocopherol (B) in *db/db* and WT mice exposed to air or ozone. Results are expressed as described in Fig 4. n = 8/group * q<0.05 versus exposure-matched WT mice. # q<0.05 versus genotype-matched air exposed mice.

There were no changes in other measured biochemical markers of oxidative stress (e.g. methionine sulfoxide, dimethylarginine (SDMA + ADMA), cysteine-glutathione disulfide, and 13-HODE + 9-HODE) with either O_3_ or obesity (see S1 Table). However, the antioxidants, ascorbate (vitamin C) (Fig 8A) and alpha-tocopherol (vitamin E) (Fig 8B) were changed. In particular, two-way ANOVA indicated an effect of obesity on both ascorbate and alpha-tocopherol: in obese mice, lung ascorbate was increased and alpha-tocopherol was decreased regardless of exposure status.

### Bacterial-mammalian co-metabolites

There are differences in the metabolomic profile of tissues harvested from germ free versus conventionally housed mice and from antibiotic-treated versus control mice [51–54]. These data indicate that bacteria are required for the generation of certain metabolites present in mammalian tissues. Such metabolites are termed bacterial-mammalian co-metabolites. We have observed reductions in the pulmonary response to O_3_ in mice after administration of antibiotics, suggesting a role for the microbiome in responses to O_3_ [55]. There are also differences in the gut microbiomes of obese and lean mice (reviewed in [56]). Consequently, we examined bacterial-mammalian co-metabolites in our data set. Twelve such metabolites [51,57–60] were identified (Table 5). Note that propionylcarnitine and butyrlcarnitine are the carnitine derivatives of propionate and butyrate, short chain fatty acids that are products of bacterial metabolism of dietary fiber. Remarkably, every one of these metabolites was significantly altered by obesity, by O_3_, or the combination of obesity and O_3_ (Table 5). These data support the hypothesis that some of the observed differences in the lung metabolomes of obese and lean mice and their changes upon exposure to O_3_ may derive from differences in their microbiomes.

**Table 5:** Effect of obesity and ozone exposure on lung microbial-mammalian co-metabolites.

Microbiome related metabolites	*Db/db*/WT Air	*Dd/db*/WT Ozone	Ozone/Air WT	Ozone/Air *Db/db*
2-aminobutyrate	0.73	0.51*	2.52#	1.75
p-cresol sulfate	0.95	0.27*	4.17#	1.17
benzoate	1.01	0.97	1.71#	1.64#
trigonelline	1.08	1.55*	0.57#	0.82
equol sulfate	0.59	4.62*	0.41	3.19
3-indoxyl sulfate	0.64	1.97*	0.64	1.98
5-aminovalerate	1.03	1.60*	0.71	1.10
phenol sulfate	1.56*	3.86*	0.63	1.56
pipecolate	1.48*	2.04*	0.93	1.28
hippurate	1.66*	2.76*	0.61	1.02
propionylcarnitine	0.64*	0.93	0.73	1.06
butyrylcarnitine	0.74*	0.87	0.84	0.99

Results are the ratio of mean metabolite scaled peak areas in *db/db* versus wildtype (WT) mice exposed to air or ozone or in ozone versus air exposed WT or *db/db* mice.

* q<0.05 versus WT;

^#^ q<0.05 versus air exposed mice of the same genotype. n = 8/group

## Discussion

Both obesity and O_3_ had substantial effects on the lung metabolome (Tables 1–3). There were also differences in the impact of O_3_ in obese and lean mice. For example, O_3_ had differential effects on lipid and BCAA metabolism (Figs 4 and 5 and Tables 2–4), and on hormones that impact these metabolic pathways (Fig 6). There were also differential effects of O_3_ on glutathione metabolism and other markers of oxidative stress in obese versus lean mice (Figs 7 and 8). Finally, both obesity and O_3_ had substantial effects on microbial-mammalian co-metabolites (Table 5), suggesting that obesity-related changes in the gut microbiome may be impacting responses to O_3._

Before discussing these data, it is important to note several caveats related to the results presented here. First, we examined metabolites in the lung. We did so because we were interested in the possibility that the effects of certain metabolites might be altering lung responses to O_3_ in obesity. Thus, it was important to know whether these metabolites changed within the lung. However, there are marked systemic effects of both obesity [19–21] and O_3_ [23,24] on metabolism, and we cannot necessarily conclude that observed changes in various lung metabolites were the result of altered metabolism *within the lung*. Blood was flushed from the lungs prior to tissue harvest, so the observed changes do not simply reflect obesity-related differences in the blood within the lungs. However, moieties in the blood can diffuse into the extracellular fluid (ECF) in the lung, and this process may be enhanced following O_3_ exposure, which increases the permeability of the alveolar/capillary barrier [61]. Consequently, the observed increases in total lung carbohydrate and lipid metabolites in *db/db* mice (Figs 2 and 3) may be the result of increases in these biochemicals in the lung ECF stemming from systemic alterations in metabolism that affect these metabolites.

Second, we studied only female mice. We did so because increases in asthma prevalence with BMI are stronger in women than men [62], and because obesity-related effects on the response to O_3_ are greater in female than male human subjects [13]. However, sex differences in the serum and urine metabolomic profiles of male and female obese mice have been reported [21]. Thus, it is possible that obesity would also have different effects on the metabolomic response to O_3_ in male and female mice. Indeed, others have reported that 17β-estradiol increases the production of GSSG in cultured alveolar epithelial cells exposed to O_3_, indicating effects of sex hormones on oxidative stress [63].

Third, our study was primarily descriptive in nature. It was performed to identify metabolites that might be contributing to the innate AHR or the augmented O_3_-induced AHR observed in obese mice. Nevertheless, there may be important mechanistic implications of our results: many of the metabolites we identified as being altered by obesity and/or O_3_ have effects that may contribute to AHR. For example, several cholesterol metabolites, including 7-α- and 7- β-hydroxycholesterol were increased in lungs of obese mice (S1 Table). These moieties are precursors for the production of 7-α 27-hydroxycholesterol and 7-β-27-hydroxycholesterol, ligands for a transcription factor RORγt that controls the production of innate lymphoid cells type 3 (ILC3) [64]. IL-17A^+^ ILC3s are increased in lungs of obese versus lean mice and contribute to the innate AHR of these mice [65]. Elevations in fatty acids in the lungs of *db/db* mice (Fig 3) would also be expected to increase the ability of ILC2s to produce type 2 cytokines [66], and we have reported that IL-33 causes ILC2 activation and type 2 cytokine release are important for obesity-related increases in the response to O_3_[16,17]. Additionally, the ketone, BHBA, was higher in the lungs of obese versus lean mice (Fig 3). Milner et al [22] reported that lung BHBA correlated positively with the number of T regulatory cells (Tregs) in the lungs during influenza virus infection in lean mice but inversely in obese mice. Whether Tregs impact pulmonary responses to acute O_3_ has not yet been established. Finally receptors for lactate (HCA1/GPR81), for many fatty acids (GPR40, GPR41, GPR43, GPR84, and GPR120), for BHBA (HCA2/GPR109A), and for other lipid moieties [67,68] exist. Consequently, the observation that these moieties are increased not only in the blood [20], but also in the lung (Figs 2 and 3), suggests that obesity-related changes in these biochemicals could impact airway responsiveness via direct activation of these receptors. Saturated fatty acids like lauric acid and palmitic acid, which were increased in lungs of obese mice (Fig 3), also have the capacity to stimulate the innate immune system by activating pattern recognition molecules and the NLRP3 inflammasome[69].

O_3_ caused substantial reductions in choline-containing lysophospholipids, especially in lean mice (Tables 2–4). Changes in phospholipids and/or lysolipids are also observed in the lungs after PM2.5 exposure [30], and after allergen sensitization and challenge [70] suggesting that these changes may represent a common response to lung injury and/or inflammation. Reductions in lysolipids after O_3_ in WT mice could be the result of reduced production from membrane phospholipids resulting from decreases in phospholipase activity. However, examination of published microarray data from C57BL/6 mice exposed in the same manner as we did (GSE38014) indicates that pulmonary mRNA abundance of most phospholipases is either increased or unchanged after O_3_. Choline containing phosphoplipids make up the majority of the phospholipids in surfactant [36] and O_3_ causes oxidation of surfactant phospholipids with consequent loss of their surface active properties [71]. Hence, it is also possible that reductions in lysolipids after O_3_ reflect their increased incorporation into surfactant phospholipids in order to replete these lipids after O_3_-induced degradation. If so, greater reductions in lysolipids in lean than obese mice after O_3_ (Tables 3 and 4), would be expected to result in less loss of surfactant function in lean than obese mice. Consistent with this prediction, we have previously reported changes in the pressure-volume curve of the lung consistent with loss of surfactant function in obese but not lean mice after O_3_ exposure [17]. LysoPC acyltransferases (LPCATs), enzymes involved in conversion of choline containing lyso lipids into phospholipids, are increased in a mouse model of sepsis in conjunction with reductions in lysophospholipids [72]. Similarly, LPCAT3 is increased after O_3_ exposure in WT mice (GSE38014) but is unchanged in *db/db* mice after O_3_ exposure, and might account for the reductions in lysolipids observed in the WT mice.

We also observed differential effects of O_3_ on the substrates used for energy production in the lungs of *db/db* and WT mice (Figs 4 and 5). In particular, O_3_ caused reductions in most BCAA metabolites in lean mice but had no effect in obese mice (Fig 4), suggesting increased reliance upon BCAA catabolism for energy in lean but not obese mice exposed to O_3_. Instead, the obese mice demonstrated increased reliance upon β-oxidation for energy after O_3_ exposure: long chain acylcarnitines were reduced after O_3_ exposure, particularly in obese mice, even though these mice had elevations in the lung fatty acids from which these acylcarnitines are derived (Fig 5). Changes in lung β-oxidation also occur following exposure to another inhaled pollutant, acrolein [26]. In particular, mice that are resistant to the effects of acrolein have evidence of increased β-oxidation, whereas sensitive mice have impaired β-oxidation.

As discussed above, greater O_3_-induced increases in corticosterone in obese than lean mice (Fig 6A) may account for the different effects of O_3_ on β-oxidation (Fig 5) and BCAA metabolism (Fig 4). Consistent with this hypothesis, O_3_ exposure also increases serum corticosterone in rats, many of the metabolomic changes induced by O_3_ in rats are attenuated in adrenalectomized rats [23,25]. Differences in insulin (Fig 6B) could also account for obesity-related differences in the effects of O_3_ on BCAA metabolism (Fig 4).

Our results support the hypothesis that lung oxidative stress was greater in obese than lean mice, especially after O_3_ exposure (Figs 7 and 8). First, the redox-regulated gene, *Gclc*, increased to a greater extent in obese than lean mice after O_3_ exposure. Second, lung GSSG was increased in obese versus lean mice after O_3_ exposure. Reductions in the antioxidant, alpha tocopherol, in lungs of obese versus lean mice may have contributed to the differences in oxidative stress. These observations are consistent with previous observations in lungs of obese human subjects [47].

To our knowledge, this is the first report of the impact of acute O_3_ exposure on the *lung* metabolome. However, as discussed above, others have reported the effect of O_3_ on the *serum* metabolome of lean rats [23]. One of the key observations in that study was that acute O_3_ exposure causes lipolysis within adipose tissue leading to increases in circulating fatty acids. In contrast, we did not observe substantial changes in lung fatty acids in either obese or lean mice after O_3_ exposure (S1 Table), though increases in some monoglycerides were observed in lean mice. Miller *et al* [23] also reported increases in BCAAs and their metabolites in lean rats exposed to O_3_, whereas we saw reductions (Fig 4). It is possible that the differences lie in the tissue examined—lung in our study versus serum in that of Miller *et al*. However, another key difference between the two studies is the time point at which these changes were noted (24 hours post O_3_ in our study versus immediately post O_3_ in the study of Miller *et al*). In this context, it is important to note that in the study of Miller *et al* [23] many of the metabolomic changes noted had substantially resolved by 18 hours after cessation of O_3_ exposure.

Of the 12 bacterial-mammalian co-metabolites identified in lungs of the mice in this study, each one was affected by obesity, by O_3_ exposure, or by the combination of these two factors (Table 5), suggesting a possible role for the microbiome in obesity-related differences in the response to O_3_. That obesity might impact metabolites of bacterial origin is not unexpected. Changes in the gut microbiome are observed both in genetically obese mice and in mice with diet-induced obesity, and obesity also impacts the human gut microbiome (see [56] for review). Gut bacteria-derived metabolites can enter the circulation and diffuse into the lungs. That O_3_ also affected these metabolites is somewhat more surprising. However, generation of many of the bacterial-mammalian co-metabolites identified (Table 5) requires a metabolic step that occurs in the liver. In rodents, acute O_3_ exposure has substantial effects on gene expression within the liver [23] and could thus impact the generation of these metabolites. Hepatic steatosis is common in obesity [73] and could alter the effect of O_3_ on the liver.

Whether or not these bacterial-mammalian co-metabolites contribute to the functional and inflammatory changes observed in the lungs after O_3_ exposure and/or the effect of obesity on these responses to O_3_ [16,17] remains to be established. However, it is increasingly appreciated that other bacterially derived metabolites contribute to pathological processes not only within the gut but also within the heart, the immune system, and the nervous system [18,74,75]. Indeed, the microbiome has already been shown to play a role in pulmonary responses to allergen [76]. Importantly, a preliminary report from our lab indicated that O_3_-induced airway hyperresponsiveness was reduced in mice treated with a cocktail of antibiotics, indicating a role for the microbiome in responses to O_3_ [55].

## Conclusion

The metabolomic profile of the lung was fundamentally altered in obesity and with O_3_ exposure. Obesity caused changes in carbohydrates and lipids in the lungs. O_3_ caused differential effects on lung lysolipids and also induced an increased reliance upon BCAA for energy production in lungs of lean mice and an increased reliance upon fatty acids for energy in obese mice, possibly as a result of greater O_3_-induced increases in corticosterone in the obese mice. Together, these metabolomic changes may have the capacity to promote the asthma-like phenotype observed in obese mice. We have previously reported that both IL-33 [16] and TNF [17] play a role in the effects of obesity on O_3_-induced AHR. Consequently, it is conceivable that these cytokines contribute to the metabolomic changes observed here. Finally, the marked effects of both obesity and O_3_ on bacterial mammalian co-metabolites also suggest a role for the microbiome in the effects of obesity on the lung.

## Supporting information

S1 Table (DOCX)

S1 Method (DOCX)

S1 Fig (DOCX)
