# Supplementary material for: Effect of acute ozone exposure on the lung metabolomes of obese and lean mice
Source: PLoS One. 2017 Jul 13;12(7):e0181017. doi: 10.1371/journal.pone.0181017 (PMC5509247; doi:10.1371/journal.pone.0181017)
Supplement: S1 Table — (DOCX) [file pone.0181017.s001.docx]

**S1 Table**

|  |  |  |  |  |  |  |  |  |  |  |  |  |  |  |  |  |  |  |  |  |  |  |  |  |
| --- | --- | --- | --- | --- | --- | --- | --- | --- | --- | --- | --- | --- | --- | --- | --- | --- | --- | --- | --- | --- | --- | --- | --- | --- |
|  |  |  |  |  |  |  |  |  |  |  |  |  |  |  |  |  |  |  |  |  |  |  |  |  |
|  |  |  |  | **ANOVA Contrasts** | | | |  | | | **Statistical Values** | | | | | | | | | | | | | |
|  |  |  |  | **db/db  WT** | | **Ozone  Ambient** | |  |  |  | **ANOVA Contrasts** | | | | | | | | **Two-Way ANOVA** | | | | | |
|  | **Super Pathway** | **Sub Pathway** | **Biochemical Name** |  |  |  |  | **Genotype  Main Effect** | **Treatment  Main Effect** | **Genotype: Treatment  Interaction** | **db/db Ambient / WT Ambient** | | **db/db Ozone / WT Ozone** | | **WT Ozone / WT Ambient** | | **db/db Ozone / db/db Ambient** | | **Genotype Main Effect** | | **Treatment Main Effect** | | **Genotype:Treatment Interaction** | |
|  |  |  |  | **Ambient** | **Ozone** | **WT** | **db/db** |  |  |  | ***p*-value** | ***q*-value** | ***p*-value** | ***q*-value** | ***p*-value** | ***q*-value** | ***p*-value** | ***q*-value** | ***p*-value** | ***q*-value** | ***p*-value** | ***q*-value** | ***p*-value** | ***q*-value** |
|  | Amino acid | Glycine, serine and threonine metabolism | glycine | 1.20 | **1.36** | 0.89 | 1.01 |  |  |  | 0.15 | 0.10 | 0.01 | 0.02 | 0.34 | 0.53 | 0.85 | 0.87 | 0.01 | 0.01 | 0.59 | 0.47 | 0.42 | 0.78 |
|  |  |  | sarcosine (N-Methylglycine) | **1.34** | 1.18 | 0.94 | 0.83 |  |  |  | 0.05 | 0.05 | 0.34 | 0.13 | 0.96 | 0.84 | 0.28 | 0.64 | 0.04 | 0.02 | 0.42 | 0.42 | 0.47 | 0.80 |
|  |  |  | N-acetylglycine | **1.21** | 1.04 | 1.23 | 1.06 |  |  |  | 0.09 | 0.07 | 0.67 | 0.22 | 0.11 | 0.28 | 0.74 | 0.85 | 0.14 | 0.05 | 0.17 | 0.26 | 0.37 | 0.77 |
|  |  |  | serine | 1.10 | **1.27** | 1.13 | **1.30** |  |  |  | 0.37 | 0.17 | 0.09 | 0.05 | 0.26 | 0.44 | 0.05 | 0.31 | 0.07 | 0.03 | 0.03 | 0.08 | 0.54 | 0.80 |
|  |  |  | homoserine | 0.88 | 1.14 | 0.92 | 1.18 |  |  |  | 0.52 | 0.20 | 0.33 | 0.13 | 0.71 | 0.75 | 0.21 | 0.54 | 0.81 | 0.23 | 0.53 | 0.46 | 0.25 | 0.75 |
|  |  |  | threonine | 0.91 | 1.05 | 1.06 | **1.23** |  |  |  | 0.54 | 0.20 | 0.58 | 0.19 | 0.43 | 0.60 | 0.06 | 0.32 | 0.96 | 0.26 | 0.06 | 0.13 | 0.41 | 0.78 |
|  |  |  | N-acetylthreonine | 0.98 | 1.08 | 0.99 | 1.09 |  |  |  | 0.87 | 0.27 | 0.57 | 0.19 | 0.83 | 0.82 | 0.61 | 0.80 | 0.77 | 0.22 | 0.83 | 0.58 | 0.61 | 0.80 |
|  |  |  | betaine | 0.87 | 1.11 | 0.83 | 1.06 |  |  |  | 0.56 | 0.21 | 0.65 | 0.21 | 0.31 | 0.50 | 1.00 | 0.90 | 0.93 | 0.25 | 0.47 | 0.43 | 0.47 | 0.80 |
|  |  | Alanine and aspartate metabolism | aspartate | **0.72** | 0.93 | 0.86 | 1.11 |  |  |  | 0.07 | 0.06 | 0.79 | 0.25 | 0.44 | 0.60 | 0.42 | 0.75 | 0.14 | 0.06 | 0.98 | 0.62 | 0.27 | 0.75 |
|  |  |  | asparagine | 0.99 | 1.12 | **1.29** | **1.46** |  |  |  | 0.83 | 0.26 | 0.45 | 0.16 | 0.07 | 0.22 | 0.02 | 0.24 | 0.49 | 0.15 | 0.01 | 0.02 | 0.70 | 0.81 |
|  |  |  | beta-alanine | 0.81 | 1.08 | 0.83 | 1.11 |  |  |  | 0.31 | 0.15 | 0.65 | 0.21 | 0.51 | 0.63 | 0.41 | 0.75 | 0.68 | 0.20 | 0.91 | 0.60 | 0.30 | 0.75 |
|  |  |  | 3-ureidopropionate | 1.14 | 1.24 | **0.55** | **0.60** |  |  |  | 0.21 | 0.12 | 0.39 | 0.14 | 0.07 | 0.21 | 0.03 | 0.25 | 0.14 | 0.05 | 0.01 | 0.02 | 0.77 | 0.83 |
|  |  |  | alanine | 1.14 | **1.34** | 1.12 | **1.32** |  |  |  | 0.28 | 0.14 | 0.03 | 0.03 | 0.31 | 0.50 | 0.03 | 0.27 | 0.02 | 0.01 | 0.03 | 0.07 | 0.41 | 0.78 |
|  |  |  | N-acetylalanine | **1.27** | **1.34** | 0.97 | 1.02 |  |  |  | 0.02 | 0.02 | 0.01 | 0.01 | 0.81 | 0.81 | 0.91 | 0.87 | 0.00 | 0.00 | 0.93 | 0.60 | 0.81 | 0.83 |
|  |  |  | N-acetylaspartate (NAA) | **1.40** | 1.27 | 0.79 | 0.71 |  |  |  | 0.07 | 0.05 | 0.26 | 0.11 | 0.49 | 0.63 | 0.16 | 0.47 | 0.04 | 0.02 | 0.14 | 0.24 | 0.60 | 0.80 |
|  |  | Glutamate metabolism | glutamate | 0.78 | 0.92 | 0.91 | 1.07 |  |  |  | 0.12 | 0.08 | 0.73 | 0.23 | 0.53 | 0.63 | 0.54 | 0.78 | 0.18 | 0.07 | 0.99 | 0.62 | 0.38 | 0.78 |
|  |  |  | glutamine | 1.13 | 0.97 | **1.47** | **1.26** |  |  |  | 0.26 | 0.13 | 0.86 | 0.26 | 0.00 | 0.02 | 0.06 | 0.32 | 0.51 | 0.16 | 0.00 | 0.01 | 0.36 | 0.77 |
|  |  |  | gamma-aminobutyrate (GABA) | **1.77** | **1.90** | 0.87 | 0.94 |  |  |  | 0.02 | 0.02 | 0.00 | 0.01 | 0.43 | 0.60 | 0.87 | 0.87 | 0.00 | 0.00 | 0.50 | 0.45 | 0.65 | 0.81 |
|  |  | Histidine metabolism | histidine | 0.91 | 0.90 | 1.21 | 1.20 |  |  |  | 0.60 | 0.21 | 0.55 | 0.19 | 0.15 | 0.33 | 0.18 | 0.49 | 0.43 | 0.14 | 0.05 | 0.12 | 0.95 | 0.86 |
|  |  | Lysine metabolism | lysine | 0.95 | 0.89 | **1.27** | 1.20 |  |  |  | 0.95 | 0.28 | 0.38 | 0.14 | 0.02 | 0.10 | 0.12 | 0.44 | 0.50 | 0.16 | 0.01 | 0.03 | 0.56 | 0.80 |
|  |  |  | 2-aminoadipate | **0.54** | **0.58** | 0.71 | **0.76** |  |  |  | 0.01 | 0.01 | 0.00 | 0.00 | 0.14 | 0.31 | 0.06 | 0.32 | 0.00 | 0.00 | 0.02 | 0.06 | 0.76 | 0.83 |
|  |  |  | pipecolate | **1.48** | **2.04** | 0.93 | **1.28** |  |  |  | 0.01 | 0.01 | 0.00 | 0.00 | 0.68 | 0.72 | 0.07 | 0.35 | 0.00 | 0.00 | 0.32 | 0.37 | 0.12 | 0.75 |
|  |  | Phenylalanine & tyrosine metabolism | phenylalanine | **1.25** | **1.22** | 0.97 | 0.95 |  |  |  | 0.04 | 0.04 | 0.08 | 0.05 | 0.94 | 0.83 | 0.66 | 0.84 | 0.01 | 0.01 | 0.72 | 0.53 | 0.80 | 0.83 |
|  |  |  | p-cresol sulfate | 0.95 | **0.27** | **4.17** | 1.17 |  |  |  | 0.85 | 0.26 | 0.00 | 0.00 | 0.00 | 0.00 | 0.57 | 0.79 | 0.00 | 0.00 | 0.00 | 0.00 | 0.00 | 0.24 |
|  |  |  | tyrosine | 1.08 | 1.17 | 0.96 | 1.04 |  |  |  | 0.30 | 0.14 | 0.15 | 0.07 | 0.92 | 0.83 | 0.74 | 0.85 | 0.08 | 0.04 | 0.86 | 0.59 | 0.76 | 0.83 |
|  |  |  | 3-(4-hydroxyphenyl)lactate | **1.39** | **1.85** | 0.85 | 1.14 |  |  |  | 0.02 | 0.02 | 0.00 | 0.00 | 0.33 | 0.52 | 0.39 | 0.73 | 0.00 | 0.00 | 0.94 | 0.61 | 0.20 | 0.75 |
|  |  |  | phenol sulfate | **1.56** | **3.86** | 0.63 | 1.56 |  |  |  | 0.02 | 0.02 | 0.00 | 0.00 | 0.36 | 0.54 | 0.10 | 0.41 | 0.00 | 0.00 | 0.59 | 0.47 | 0.07 | 0.75 |
|  |  | Tryptophan metabolism | kynurenine | **0.70** | 0.81 | 0.89 | 1.02 |  |  |  | 0.08 | 0.06 | 0.25 | 0.10 | 0.94 | 0.83 | 0.60 | 0.80 | 0.04 | 0.02 | 0.75 | 0.54 | 0.67 | 0.81 |
|  |  |  | tryptophan | 1.01 | 1.18 | 0.97 | 1.13 |  |  |  | 0.61 | 0.21 | 0.18 | 0.08 | 0.93 | 0.83 | 0.36 | 0.71 | 0.19 | 0.07 | 0.48 | 0.44 | 0.56 | 0.80 |
|  |  |  | C-glycosyltryptophan* | 1.11 | **1.24** | 0.90 | 1.01 |  |  |  | 0.29 | 0.14 | 0.04 | 0.03 | 0.39 | 0.56 | 0.86 | 0.87 | 0.03 | 0.02 | 0.62 | 0.49 | 0.46 | 0.80 |
|  |  |  | 5-hydroxyindoleacetate | 0.59 | 1.12 | 0.54 | 1.04 |  |  |  | 0.50 | 0.20 | 0.38 | 0.14 | 0.13 | 0.29 | 1.00 | 0.90 | 0.89 | 0.25 | 0.28 | 0.34 | 0.27 | 0.75 |
|  |  |  | 3-indoxyl sulfate | **0.64** | **1.97** | 0.64 | **1.98** |  |  |  | 0.09 | 0.07 | 0.00 | 0.01 | 0.12 | 0.28 | 0.00 | 0.08 | 0.34 | 0.11 | 0.27 | 0.34 | 0.00 | 0.24 |
|  |  | Valine, leucine and isoleucine metabolism | isoleucine | **1.19** | **1.30** | 0.97 | 1.06 |  |  |  | 0.08 | 0.06 | 0.02 | 0.02 | 0.91 | 0.83 | 0.66 | 0.83 | 0.01 | 0.01 | 0.82 | 0.58 | 0.69 | 0.81 |
|  |  |  | leucine | 1.11 | **1.21** | 0.98 | 1.07 |  |  |  | 0.24 | 0.13 | 0.08 | 0.05 | 0.94 | 0.83 | 0.59 | 0.80 | 0.04 | 0.02 | 0.74 | 0.54 | 0.66 | 0.81 |
|  |  |  | valine | **1.18** | **1.29** | 0.95 | 1.04 |  |  |  | 0.07 | 0.06 | 0.02 | 0.02 | 0.80 | 0.80 | 0.77 | 0.85 | 0.00 | 0.00 | 0.98 | 0.62 | 0.70 | 0.81 |
|  |  |  | 3-hydroxyisobutyrate | **1.24** | **1.65** | **0.75** | 1.00 |  |  |  | 0.07 | 0.06 | 0.00 | 0.00 | 0.07 | 0.22 | 0.94 | 0.89 | 0.00 | 0.00 | 0.18 | 0.27 | 0.21 | 0.75 |
|  |  |  | alpha-hydroxyisovalerate | **1.45** | **1.67** | 0.90 | 1.03 |  |  |  | 0.06 | 0.05 | 0.08 | 0.05 | 0.99 | 0.84 | 0.91 | 0.87 | 0.01 | 0.01 | 0.93 | 0.60 | 0.94 | 0.86 |
|  |  |  | isobutyrylcarnitine | 1.08 | **1.69** | **0.65** | 1.02 |  |  |  | 0.46 | 0.19 | 0.00 | 0.00 | 0.02 | 0.10 | 0.88 | 0.87 | 0.01 | 0.01 | 0.12 | 0.21 | 0.08 | 0.75 |
|  |  |  | 2-methylbutyrylcarnitine (C5) | 1.26 | **1.90** | **0.55** | 0.82 |  |  |  | 0.11 | 0.07 | 0.00 | 0.00 | 0.00 | 0.02 | 0.28 | 0.64 | 0.00 | 0.00 | 0.00 | 0.01 | 0.10 | 0.75 |
|  |  |  | isovalerylcarnitine | **1.38** | **2.28** | **0.50** | 0.83 |  |  |  | 0.05 | 0.05 | 0.00 | 0.00 | 0.00 | 0.02 | 0.27 | 0.62 | 0.00 | 0.00 | 0.00 | 0.01 | 0.06 | 0.75 |
|  |  |  | hydroxyisovaleroyl carnitine | **1.36** | **1.47** | **0.75** | 0.81 |  |  |  | 0.02 | 0.03 | 0.01 | 0.01 | 0.05 | 0.17 | 0.11 | 0.43 | 0.00 | 0.00 | 0.01 | 0.04 | 0.75 | 0.82 |
|  |  | Cysteine, methionine, SAM, taurine metabolism | cysteine | 0.90 | 1.39 | 0.57 | 0.88 |  |  |  | 0.44 | 0.18 | 0.15 | 0.07 | 0.25 | 0.44 | 0.63 | 0.81 | 0.12 | 0.05 | 0.25 | 0.34 | 0.63 | 0.81 |
|  |  |  | S-methylcysteine | 1.03 | 1.31 | 1.04 | 1.32 |  |  |  | 0.98 | 0.29 | 0.31 | 0.12 | 0.97 | 0.84 | 0.30 | 0.65 | 0.45 | 0.14 | 0.44 | 0.43 | 0.48 | 0.80 |
|  |  |  | methionine sulfoxide | 0.83 | 1.19 | 0.76 | 1.10 |  |  |  | 0.42 | 0.18 | 0.16 | 0.07 | 0.10 | 0.27 | 0.55 | 0.78 | 0.66 | 0.20 | 0.45 | 0.43 | 0.12 | 0.75 |
|  |  |  | hypotaurine | 1.02 | **2.64** | 0.98 | **2.54** |  |  |  | 0.84 | 0.26 | 0.00 | 0.00 | 0.95 | 0.83 | 0.00 | 0.04 | 0.01 | 0.01 | 0.01 | 0.04 | 0.01 | 0.51 |
|  |  |  | taurine | **1.33** | **1.44** | **0.81** | **0.88** |  |  |  | 0.00 | 0.00 | 0.00 | 0.00 | 0.00 | 0.02 | 0.04 | 0.30 | 0.00 | 0.00 | 0.00 | 0.00 | 0.34 | 0.76 |
|  |  |  | S-adenosylhomocysteine (SAH) | 0.96 | 1.19 | 0.95 | 1.17 |  |  |  | 0.81 | 0.26 | 0.22 | 0.09 | 0.58 | 0.66 | 0.35 | 0.71 | 0.47 | 0.15 | 0.79 | 0.57 | 0.30 | 0.75 |
|  |  |  | methionine | 1.02 | 1.11 | 1.00 | 1.10 |  |  |  | 0.69 | 0.23 | 0.24 | 0.10 | 0.86 | 0.82 | 0.34 | 0.70 | 0.27 | 0.09 | 0.42 | 0.42 | 0.58 | 0.80 |
|  |  |  | N-acetylmethionine | **1.27** | **1.30** | 1.01 | 1.03 |  |  |  | 0.07 | 0.06 | 0.03 | 0.03 | 0.99 | 0.84 | 0.71 | 0.85 | 0.01 | 0.01 | 0.80 | 0.57 | 0.78 | 0.83 |
|  |  |  | 2-hydroxybutyrate (AHB) | **1.42** | 1.29 | **1.34** | 1.21 |  |  |  | 0.03 | 0.03 | 0.30 | 0.12 | 0.05 | 0.18 | 0.47 | 0.75 | 0.02 | 0.01 | 0.06 | 0.13 | 0.36 | 0.77 |
|  |  | Urea cycle; arginine-, proline-, metabolism | dimethylarginine (SDMA + ADMA) | 1.06 | 1.11 | 1.10 | 1.15 |  |  |  | 0.41 | 0.18 | 0.29 | 0.12 | 0.20 | 0.38 | 0.13 | 0.44 | 0.19 | 0.07 | 0.05 | 0.12 | 0.87 | 0.84 |
|  |  |  | arginine | 1.01 | 1.06 | 1.01 | 1.06 |  |  |  | 0.78 | 0.25 | 0.21 | 0.09 | 0.75 | 0.78 | 0.19 | 0.52 | 0.28 | 0.09 | 0.25 | 0.34 | 0.48 | 0.80 |
|  |  |  | ornithine | 1.24 | 1.61 | 1.21 | 1.57 |  |  |  | 0.40 | 0.17 | 0.31 | 0.12 | 0.29 | 0.48 | 0.22 | 0.55 | 0.19 | 0.07 | 0.11 | 0.20 | 0.90 | 0.86 |
|  |  |  | urea | 0.94 | 1.37 | 0.63 | 0.92 |  |  |  | 0.78 | 0.25 | 0.16 | 0.07 | 0.20 | 0.37 | 0.88 | 0.87 | 0.23 | 0.08 | 0.31 | 0.37 | 0.42 | 0.78 |
|  |  |  | proline | 1.04 | **1.20** | 0.98 | 1.13 |  |  |  | 0.60 | 0.21 | 0.08 | 0.05 | 0.93 | 0.83 | 0.24 | 0.59 | 0.11 | 0.05 | 0.43 | 0.43 | 0.37 | 0.77 |
|  |  |  | 5-aminovalerate | 1.03 | **1.60** | 0.71 | 1.10 |  |  |  | 0.47 | 0.19 | 0.02 | 0.02 | 0.20 | 0.37 | 0.70 | 0.85 | 0.03 | 0.02 | 0.52 | 0.45 | 0.24 | 0.75 |
|  |  |  | citrulline | 0.96 | 0.88 | **2.08** | **1.92** |  |  |  | 0.82 | 0.26 | 0.64 | 0.21 | 0.00 | 0.00 | 0.00 | 0.04 | 0.87 | 0.24 | 0.00 | 0.00 | 0.62 | 0.81 |
|  |  |  | trans-4-hydroxyproline | 0.86 | 0.97 | 0.97 | 1.09 |  |  |  | 0.47 | 0.19 | 0.87 | 0.26 | 0.99 | 0.84 | 0.57 | 0.79 | 0.53 | 0.16 | 0.68 | 0.52 | 0.69 | 0.81 |
|  |  |  | homocitrulline | **1.29** | 1.18 | 1.26 | 1.15 |  |  |  | 0.08 | 0.06 | 0.36 | 0.14 | 0.10 | 0.27 | 0.44 | 0.75 | 0.06 | 0.03 | 0.09 | 0.18 | 0.53 | 0.80 |
|  |  |  | N-delta-acetylornithine* | **1.40** | **1.63** | 1.06 | 1.24 |  |  |  | 0.00 | 0.01 | 0.00 | 0.00 | 0.57 | 0.66 | 0.11 | 0.43 | 0.00 | 0.00 | 0.13 | 0.23 | 0.46 | 0.80 |
|  |  | Creatine metabolism | creatine | 0.94 | 0.99 | 1.07 | 1.13 |  |  |  | 0.83 | 0.26 | 0.92 | 0.27 | 0.46 | 0.61 | 0.29 | 0.64 | 0.93 | 0.25 | 0.20 | 0.29 | 0.82 | 0.83 |
|  |  | Butanoate metabolism | 2-aminobutyrate | 0.73 | **0.51** | **2.52** | 1.75 |  |  |  | 0.62 | 0.21 | 0.00 | 0.01 | 0.00 | 0.01 | 0.14 | 0.44 | 0.01 | 0.01 | 0.00 | 0.00 | 0.07 | 0.75 |
|  |  | Polyamine metabolism | 5-methylthioadenosine (MTA) | **1.40** | **1.28** | 1.10 | 1.00 |  |  |  | 0.00 | 0.00 | 0.01 | 0.02 | 0.33 | 0.52 | 0.96 | 0.89 | 0.00 | 0.00 | 0.47 | 0.43 | 0.51 | 0.80 |
|  |  |  | putrescine | 1.29 | 0.92 | **2.94** | **2.08** |  |  |  | 0.16 | 0.10 | 0.81 | 0.25 | 0.00 | 0.00 | 0.00 | 0.03 | 0.40 | 0.13 | 0.00 | 0.00 | 0.24 | 0.75 |
|  |  |  | spermidine | 0.82 | 0.91 | 1.14 | **1.27** |  |  |  | 0.16 | 0.10 | 0.35 | 0.13 | 0.18 | 0.36 | 0.07 | 0.34 | 0.10 | 0.04 | 0.03 | 0.07 | 0.73 | 0.82 |
|  |  | Glutathione metabolism | glutathione, reduced (GSH) | **0.64** | 0.79 | **1.66** | **2.03** |  |  |  | 0.07 | 0.05 | 0.19 | 0.09 | 0.00 | 0.03 | 0.00 | 0.04 | 0.03 | 0.02 | 0.00 | 0.00 | 0.69 | 0.81 |
|  |  |  | S-methylglutathione | 1.15 | **1.53** | **0.78** | 1.04 |  |  |  | 0.31 | 0.15 | 0.00 | 0.01 | 0.10 | 0.27 | 0.75 | 0.85 | 0.01 | 0.01 | 0.33 | 0.37 | 0.16 | 0.75 |
|  |  |  | 5-oxoproline | **0.74** | **0.77** | 1.26 | 1.31 |  |  |  | 0.06 | 0.05 | 0.07 | 0.05 | 0.15 | 0.33 | 0.14 | 0.44 | 0.01 | 0.01 | 0.04 | 0.10 | 0.96 | 0.86 |
|  |  |  | glutathione, oxidized (GSSG) | 1.15 | **1.37** | 0.99 | 1.18 |  |  |  | 0.16 | 0.10 | 0.01 | 0.01 | 0.98 | 0.84 | 0.20 | 0.53 | 0.01 | 0.01 | 0.36 | 0.38 | 0.37 | 0.78 |
|  |  |  | cysteine-glutathione disulfide | 0.95 | 1.48 | 0.67 | 1.04 |  |  |  | 0.55 | 0.21 | 0.10 | 0.06 | 0.43 | 0.60 | 0.78 | 0.85 | 0.11 | 0.05 | 0.72 | 0.53 | 0.45 | 0.80 |
|  |  |  | ophthalmate | **0.53** | **0.26** | **1.88** | 0.94 |  |  |  | 0.00 | 0.01 | 0.00 | 0.00 | 0.00 | 0.02 | 0.34 | 0.70 | 0.00 | 0.00 | 0.10 | 0.19 | 0.00 | 0.33 |
|  |  |  | S-lactoylglutathione | 1.09 | **3.18** | **0.40** | 1.16 |  |  |  | 0.60 | 0.21 | 0.00 | 0.00 | 0.00 | 0.02 | 0.84 | 0.87 | 0.00 | 0.00 | 0.02 | 0.07 | 0.01 | 0.51 |
|  | Peptide | Dipeptide | glycylglycine | 1.36 | 1.38 | 1.14 | 1.15 |  |  |  | 0.21 | 0.12 | 0.13 | 0.07 | 0.73 | 0.76 | 0.56 | 0.78 | 0.05 | 0.03 | 0.51 | 0.45 | 0.86 | 0.84 |
|  |  |  | glycylproline | **1.31** | 1.18 | 1.19 | 1.07 |  |  |  | 0.03 | 0.03 | 0.14 | 0.07 | 0.12 | 0.28 | 0.43 | 0.75 | 0.01 | 0.01 | 0.10 | 0.19 | 0.58 | 0.80 |
|  |  |  | glycylleucine | **1.33** | 1.20 | 1.10 | 0.98 |  |  |  | 0.03 | 0.03 | 0.14 | 0.07 | 0.66 | 0.71 | 0.75 | 0.85 | 0.01 | 0.01 | 0.93 | 0.60 | 0.60 | 0.80 |
|  |  |  | pro-hydroxy-pro | 1.10 | 1.12 | 0.97 | 0.99 |  |  |  | 0.33 | 0.15 | 0.31 | 0.12 | 0.85 | 0.82 | 0.87 | 0.87 | 0.16 | 0.06 | 0.80 | 0.57 | 0.99 | 0.86 |
|  |  |  | prolylproline | 1.05 | 1.27 | 0.91 | 1.11 |  |  |  | 0.95 | 0.29 | 0.16 | 0.07 | 0.52 | 0.63 | 0.46 | 0.75 | 0.29 | 0.10 | 0.94 | 0.61 | 0.33 | 0.76 |
|  |  | Dipeptide derivative | anserine | 0.76 | **0.55** | 1.29 | 0.93 |  |  |  | 0.25 | 0.13 | 0.05 | 0.04 | 0.59 | 0.66 | 0.75 | 0.85 | 0.03 | 0.02 | 0.87 | 0.59 | 0.54 | 0.80 |
|  |  | gamma-glutamyl | gamma-glutamylleucine | 1.05 | 1.20 | 0.82 | 0.93 |  |  |  | 0.52 | 0.20 | 0.27 | 0.11 | 0.23 | 0.41 | 0.46 | 0.75 | 0.22 | 0.08 | 0.18 | 0.26 | 0.74 | 0.82 |
|  |  |  | gamma-glutamylglycine | 0.79 | 1.00 | 0.80 | 1.01 |  |  |  | 0.58 | 0.21 | 0.85 | 0.26 | 0.52 | 0.63 | 0.93 | 0.88 | 0.80 | 0.23 | 0.69 | 0.52 | 0.60 | 0.80 |
|  |  |  | gamma-glutamylglutamate | **0.59** | 0.76 | 0.83 | 1.07 |  |  |  | 0.02 | 0.03 | 0.31 | 0.12 | 0.32 | 0.51 | 0.73 | 0.85 | 0.02 | 0.01 | 0.65 | 0.51 | 0.34 | 0.76 |
|  |  |  | gamma-glutamylglutamine | 0.81 | **0.74** | **1.59** | **1.46** |  |  |  | 0.13 | 0.09 | 0.02 | 0.02 | 0.00 | 0.02 | 0.01 | 0.11 | 0.01 | 0.01 | 0.00 | 0.00 | 0.56 | 0.80 |
|  |  |  | gamma-glutamylphenylalanine | 1.05 | 1.01 | 1.26 | 1.21 |  |  |  | 0.77 | 0.25 | 0.84 | 0.26 | 0.32 | 0.51 | 0.36 | 0.71 | 0.72 | 0.21 | 0.18 | 0.27 | 0.95 | 0.86 |
|  |  |  | gamma-glutamyltyrosine | 0.95 | 1.12 | 0.93 | 1.11 |  |  |  | 0.93 | 0.28 | 0.35 | 0.13 | 0.64 | 0.70 | 0.58 | 0.79 | 0.55 | 0.17 | 0.95 | 0.61 | 0.47 | 0.80 |
|  |  |  | gamma-glutamylthreonine* | 0.70 | 0.97 | 0.89 | 1.23 |  |  |  | 0.16 | 0.10 | 0.65 | 0.21 | 0.85 | 0.82 | 0.43 | 0.75 | 0.19 | 0.07 | 0.67 | 0.51 | 0.49 | 0.80 |
|  | Carbohydrate | Aminosugars metabolism | N-acetylglucosamine | 1.13 | 1.23 | 1.16 | 1.26 |  |  |  | 0.22 | 0.12 | 0.13 | 0.07 | 0.20 | 0.37 | 0.11 | 0.42 | 0.06 | 0.03 | 0.04 | 0.10 | 0.81 | 0.83 |
|  |  |  | erythronate* | **1.72** | **1.94** | **0.70** | 0.79 |  |  |  | 0.00 | 0.00 | 0.00 | 0.00 | 0.04 | 0.15 | 0.13 | 0.44 | 0.00 | 0.00 | 0.01 | 0.04 | 0.67 | 0.81 |
|  |  |  | N-acetylneuraminate | 0.98 | 1.01 | 0.84 | 0.87 |  |  |  | 0.86 | 0.27 | 0.68 | 0.22 | 0.35 | 0.54 | 0.48 | 0.75 | 0.68 | 0.20 | 0.25 | 0.33 | 0.87 | 0.84 |
|  |  |  | Isobar: UDP-acetylglucosamine, UDP-acetylgalactosamine | 1.14 | **1.31** | 1.08 | 1.25 |  |  |  | 0.18 | 0.11 | 0.08 | 0.05 | 0.32 | 0.51 | 0.16 | 0.47 | 0.03 | 0.02 | 0.09 | 0.18 | 0.76 | 0.83 |
|  |  | Fructose, mannose, galactose, starch, and sucrose metabolism | fructose | **2.07** | **1.80** | 0.79 | **0.69** |  |  |  | 0.00 | 0.00 | 0.01 | 0.01 | 0.77 | 0.79 | 0.05 | 0.31 | 0.00 | 0.00 | 0.11 | 0.20 | 0.23 | 0.75 |
|  |  |  | maltose | **1.66** | **1.99** | 0.92 | 1.11 |  |  |  | 0.01 | 0.02 | 0.00 | 0.00 | 0.90 | 0.83 | 0.62 | 0.81 | 0.00 | 0.00 | 0.79 | 0.57 | 0.66 | 0.81 |
|  |  |  | mannitol | **2.83** | **2.35** | 1.09 | 0.91 |  |  |  | 0.00 | 0.00 | 0.00 | 0.00 | 0.59 | 0.66 | 0.51 | 0.76 | 0.00 | 0.00 | 0.93 | 0.60 | 0.40 | 0.78 |
|  |  |  | mannose | **1.44** | **1.34** | 0.96 | 0.89 |  |  |  | 0.01 | 0.02 | 0.04 | 0.03 | 0.90 | 0.83 | 0.51 | 0.76 | 0.00 | 0.00 | 0.58 | 0.47 | 0.71 | 0.82 |
|  |  |  | mannose-1-phosphate | **1.42** | **1.38** | 1.13 | 1.10 |  |  |  | 0.05 | 0.05 | 0.04 | 0.03 | 0.62 | 0.68 | 0.53 | 0.77 | 0.01 | 0.01 | 0.43 | 0.42 | 0.93 | 0.86 |
|  |  |  | mannose-6-phosphate | 1.19 | **1.35** | 0.85 | 0.97 |  |  |  | 0.28 | 0.14 | 0.06 | 0.04 | 0.41 | 0.58 | 0.99 | 0.90 | 0.04 | 0.02 | 0.56 | 0.47 | 0.56 | 0.80 |
|  |  |  | sorbitol | **2.30** | **1.93** | 0.94 | **0.79** |  |  |  | 0.00 | 0.00 | 0.00 | 0.00 | 0.86 | 0.82 | 0.08 | 0.37 | 0.00 | 0.00 | 0.17 | 0.26 | 0.26 | 0.75 |
|  |  |  | maltotriose | **1.80** | **1.90** | 0.81 | 0.85 |  |  |  | 0.02 | 0.03 | 0.04 | 0.03 | 0.50 | 0.63 | 0.36 | 0.71 | 0.00 | 0.00 | 0.27 | 0.34 | 0.86 | 0.84 |
|  |  |  | maltopentaose | **1.98** | **1.80** | **0.46** | **0.42** |  |  |  | 0.02 | 0.02 | 0.02 | 0.02 | 0.00 | 0.02 | 0.00 | 0.06 | 0.00 | 0.00 | 0.00 | 0.00 | 0.98 | 0.86 |
|  |  | Oligosaccharide | maltotetraose | **1.92** | **2.12** | **0.71** | **0.78** |  |  |  | 0.00 | 0.00 | 0.00 | 0.00 | 0.02 | 0.08 | 0.09 | 0.40 | 0.00 | 0.00 | 0.00 | 0.02 | 0.57 | 0.80 |
|  |  | Glycolysis, gluconeogenesis, pyruvate metabolism | 1,5-anhydroglucitol (1,5-AG) | **0.23** | **0.40** | 0.70 | 1.24 |  |  |  | 0.00 | 0.00 | 0.00 | 0.00 | 0.11 | 0.28 | 0.49 | 0.76 | 0.00 | 0.00 | 0.51 | 0.45 | 0.11 | 0.75 |
|  |  |  | glycerate | **1.56** | **1.66** | 0.82 | 0.88 |  |  |  | 0.01 | 0.02 | 0.01 | 0.01 | 0.52 | 0.63 | 0.51 | 0.76 | 0.00 | 0.00 | 0.36 | 0.38 | 0.99 | 0.86 |
|  |  |  | glucose-6-phosphate (G6P) | 1.20 | **1.32** | 0.83 | 0.91 |  |  |  | 0.23 | 0.12 | 0.08 | 0.05 | 0.33 | 0.52 | 0.70 | 0.85 | 0.04 | 0.02 | 0.34 | 0.37 | 0.67 | 0.81 |
|  |  |  | glucose | **2.62** | **2.97** | 0.87 | 0.99 |  |  |  | 0.00 | 0.00 | 0.00 | 0.00 | 0.95 | 0.83 | 0.81 | 0.87 | 0.00 | 0.00 | 0.83 | 0.58 | 0.90 | 0.86 |
|  |  |  | fructose-6-phosphate | 1.30 | **1.36** | 0.79 | 0.83 |  |  |  | 0.18 | 0.11 | 0.06 | 0.04 | 0.18 | 0.36 | 0.41 | 0.75 | 0.03 | 0.02 | 0.13 | 0.23 | 0.70 | 0.81 |
|  |  |  | Isobar: fructose 1,6-diphosphate, glucose 1,6-diphosphate, myo-inositol 1,4 or 1,3-diphosphate | **1.23** | **1.34** | 0.87 | 0.94 |  |  |  | 0.05 | 0.05 | 0.01 | 0.01 | 0.20 | 0.38 | 0.63 | 0.81 | 0.00 | 0.00 | 0.22 | 0.30 | 0.57 | 0.80 |
|  |  |  | 3-phosphoglycerate | **1.30** | **1.33** | 0.92 | 0.94 |  |  |  | 0.02 | 0.02 | 0.02 | 0.02 | 0.55 | 0.65 | 0.45 | 0.75 | 0.00 | 0.00 | 0.34 | 0.37 | 0.91 | 0.86 |
|  |  |  | phosphoenolpyruvate (PEP) | **1.44** | 1.34 | 0.97 | 0.90 |  |  |  | 0.06 | 0.05 | 0.13 | 0.07 | 0.86 | 0.82 | 0.59 | 0.80 | 0.02 | 0.01 | 0.62 | 0.49 | 0.80 | 0.83 |
|  |  |  | lactate | **1.43** | **1.58** | 1.04 | 1.15 |  |  |  | 0.02 | 0.02 | 0.01 | 0.01 | 0.61 | 0.67 | 0.37 | 0.72 | 0.00 | 0.00 | 0.32 | 0.37 | 0.79 | 0.83 |
|  |  | Nucleotide sugars, pentose metabolism | 6-phosphogluconate | **1.85** | **2.44** | **0.67** | 0.89 |  |  |  | 0.01 | 0.01 | 0.00 | 0.00 | 0.09 | 0.26 | 0.75 | 0.85 | 0.00 | 0.00 | 0.16 | 0.25 | 0.32 | 0.76 |
|  |  |  | arabitol | 0.99 | 1.20 | 0.70 | 0.84 |  |  |  | 0.98 | 0.29 | 0.45 | 0.16 | 0.22 | 0.40 | 0.61 | 0.80 | 0.58 | 0.18 | 0.22 | 0.31 | 0.61 | 0.80 |
|  |  |  | sedoheptulose-7-phosphate | 1.07 | 0.98 | 1.03 | 0.94 |  |  |  | 0.68 | 0.23 | 0.72 | 0.23 | 0.99 | 0.84 | 0.96 | 0.89 | 0.59 | 0.18 | 0.98 | 0.62 | 0.96 | 0.86 |
|  |  |  | ribose | 1.08 | **1.44** | 0.80 | 1.06 |  |  |  | 0.56 | 0.21 | 0.06 | 0.04 | 0.36 | 0.54 | 0.67 | 0.84 | 0.08 | 0.04 | 0.72 | 0.53 | 0.34 | 0.76 |
|  |  |  | ribose 5-phosphate | **0.65** | 1.02 | 0.88 | 1.38 |  |  |  | 0.06 | 0.05 | 0.93 | 0.28 | 0.78 | 0.80 | 0.12 | 0.44 | 0.16 | 0.06 | 0.36 | 0.38 | 0.20 | 0.75 |
|  |  |  | ribulose | 1.12 | **1.62** | 0.71 | 1.03 |  |  |  | 0.59 | 0.21 | 0.09 | 0.05 | 0.38 | 0.55 | 0.74 | 0.85 | 0.11 | 0.05 | 0.69 | 0.52 | 0.39 | 0.78 |
|  |  |  | Isobar: ribulose 5-phosphate, xylulose 5-phosphate | 0.82 | 1.02 | 0.87 | 1.09 |  |  |  | 0.34 | 0.16 | 0.73 | 0.23 | 0.51 | 0.63 | 0.52 | 0.76 | 0.66 | 0.20 | 1.00 | 0.62 | 0.36 | 0.77 |
|  |  |  | UDP-glucuronate | **1.34** | **1.44** | 1.11 | 1.19 |  |  |  | 0.02 | 0.02 | 0.01 | 0.01 | 0.26 | 0.44 | 0.13 | 0.44 | 0.00 | 0.00 | 0.07 | 0.14 | 0.78 | 0.83 |
|  |  |  | xylitol | **1.52** | **2.02** | 0.79 | 1.05 |  |  |  | 0.01 | 0.02 | 0.00 | 0.00 | 0.10 | 0.27 | 0.69 | 0.85 | 0.00 | 0.00 | 0.37 | 0.39 | 0.15 | 0.75 |
|  |  |  | xylonate | **1.35** | 1.20 | **0.74** | **0.65** |  |  |  | 0.02 | 0.02 | 0.16 | 0.08 | 0.03 | 0.14 | 0.00 | 0.07 | 0.01 | 0.01 | 0.00 | 0.00 | 0.43 | 0.79 |
|  |  | Nucleotide sugars | UDP-galactose | 0.93 | **1.25** | 0.82 | 1.10 |  |  |  | 0.77 | 0.25 | 0.09 | 0.05 | 0.19 | 0.37 | 0.49 | 0.76 | 0.31 | 0.11 | 0.66 | 0.51 | 0.16 | 0.75 |
|  | Energy | Krebs cycle | citrate | **1.43** | **1.37** | 1.06 | 1.01 |  |  |  | 0.01 | 0.02 | 0.03 | 0.03 | 0.51 | 0.63 | 0.84 | 0.87 | 0.00 | 0.00 | 0.54 | 0.46 | 0.74 | 0.82 |
|  |  |  | succinylcarnitine | 1.15 | **1.31** | 0.90 | 1.03 |  |  |  | 0.23 | 0.13 | 0.03 | 0.03 | 0.51 | 0.63 | 0.68 | 0.85 | 0.02 | 0.01 | 0.86 | 0.59 | 0.45 | 0.80 |
|  |  |  | fumarate | 1.22 | **1.55** | 0.97 | 1.24 |  |  |  | 0.19 | 0.11 | 0.02 | 0.02 | 1.00 | 0.84 | 0.25 | 0.60 | 0.01 | 0.01 | 0.41 | 0.42 | 0.41 | 0.78 |
|  |  |  | malate | 1.07 | 1.19 | 1.07 | 1.20 |  |  |  | 0.38 | 0.17 | 0.13 | 0.07 | 0.49 | 0.63 | 0.18 | 0.50 | 0.10 | 0.04 | 0.15 | 0.25 | 0.65 | 0.81 |
|  |  | Oxidative phosphorylation | phosphate | 1.03 | 1.04 | 0.99 | 1.01 |  |  |  | 0.49 | 0.19 | 0.29 | 0.12 | 0.84 | 0.82 | 0.86 | 0.87 | 0.22 | 0.08 | 0.98 | 0.62 | 0.79 | 0.83 |
|  |  |  | pyrophosphate (PPi) | 0.76 | **0.62** | 0.82 | 0.68 |  |  |  | 0.43 | 0.18 | 0.08 | 0.05 | 0.87 | 0.82 | 0.25 | 0.60 | 0.08 | 0.03 | 0.35 | 0.38 | 0.49 | 0.80 |
|  | Lipid | Essential fatty acid | linoleate (18:2n6) | **1.68** | **1.37** | **1.23** | 1.01 |  |  |  | 0.00 | 0.00 | 0.03 | 0.03 | 0.09 | 0.26 | 0.82 | 0.87 | 0.00 | 0.00 | 0.17 | 0.26 | 0.29 | 0.75 |
|  |  |  | linolenate [alpha or gamma; (18:3n3 or 6)] | **2.38** | **2.22** | 1.03 | 0.96 |  |  |  | 0.00 | 0.00 | 0.00 | 0.00 | 0.51 | 0.63 | 0.91 | 0.87 | 0.00 | 0.00 | 0.70 | 0.52 | 0.59 | 0.80 |
|  |  |  | dihomo-linolenate (20:3n3 or n6) | **1.55** | **1.48** | 1.00 | 0.96 |  |  |  | 0.00 | 0.00 | 0.00 | 0.00 | 0.88 | 0.82 | 0.85 | 0.87 | 0.00 | 0.00 | 0.98 | 0.62 | 0.81 | 0.83 |
|  |  |  | eicosapentaenoate (EPA; 20:5n3) | **2.48** | **2.37** | 1.08 | 1.03 |  |  |  | 0.00 | 0.00 | 0.00 | 0.00 | 0.37 | 0.55 | 0.87 | 0.87 | 0.00 | 0.00 | 0.45 | 0.43 | 0.60 | 0.80 |
|  |  |  | docosapentaenoate (n3 DPA; 22:5n3) | **2.36** | **1.97** | 1.17 | 0.98 |  |  |  | 0.00 | 0.00 | 0.00 | 0.00 | 0.11 | 0.27 | 0.91 | 0.87 | 0.00 | 0.00 | 0.29 | 0.35 | 0.22 | 0.75 |
|  |  |  | docosapentaenoate (n6 DPA; 22:5n6) | 1.14 | 0.94 | 1.17 | 0.97 |  |  |  | 0.29 | 0.14 | 0.58 | 0.20 | 0.19 | 0.37 | 0.78 | 0.85 | 0.71 | 0.21 | 0.45 | 0.43 | 0.26 | 0.75 |
|  |  |  | docosahexaenoate (DHA; 22:6n3) | **1.47** | 1.05 | **1.53** | 1.09 |  |  |  | 0.00 | 0.00 | 0.70 | 0.23 | 0.00 | 0.01 | 0.38 | 0.72 | 0.01 | 0.01 | 0.00 | 0.01 | 0.03 | 0.74 |
|  |  | Medium chain fatty acid | caproate (6:0) | 1.03 | **1.39** | 0.82 | 1.11 |  |  |  | 0.58 | 0.21 | 0.05 | 0.04 | 0.40 | 0.58 | 0.52 | 0.76 | 0.08 | 0.03 | 0.90 | 0.60 | 0.30 | 0.75 |
|  |  |  | caprate (10:0) | **1.22** | 1.11 | **1.18** | 1.08 |  |  |  | 0.03 | 0.03 | 0.21 | 0.09 | 0.05 | 0.18 | 0.32 | 0.67 | 0.02 | 0.01 | 0.04 | 0.09 | 0.46 | 0.80 |
|  |  |  | laurate (12:0) | **2.04** | **1.64** | **1.21** | 0.97 |  |  |  | 0.00 | 0.00 | 0.00 | 0.00 | 0.06 | 0.18 | 0.97 | 0.89 | 0.00 | 0.00 | 0.18 | 0.27 | 0.16 | 0.75 |
|  |  | Long chain fatty acid | myristate (14:0) | **2.24** | **1.99** | 1.07 | 0.95 |  |  |  | 0.00 | 0.00 | 0.00 | 0.00 | 0.50 | 0.63 | 0.91 | 0.87 | 0.00 | 0.00 | 0.58 | 0.47 | 0.69 | 0.81 |
|  |  |  | myristoleate (14:1n5) | **3.95** | **3.80** | 1.09 | 1.05 |  |  |  | 0.00 | 0.00 | 0.00 | 0.00 | 0.40 | 0.58 | 0.55 | 0.78 | 0.00 | 0.00 | 0.31 | 0.37 | 0.86 | 0.84 |
|  |  |  | pentadecanoate (15:0) | **1.47** | 0.99 | 1.23 | 0.83 |  |  |  | 0.02 | 0.03 | 0.91 | 0.27 | 0.11 | 0.27 | 0.40 | 0.74 | 0.11 | 0.05 | 0.57 | 0.47 | 0.08 | 0.75 |
|  |  |  | palmitate (16:0) | **1.71** | **1.57** | 1.06 | 0.97 |  |  |  | 0.00 | 0.00 | 0.00 | 0.00 | 0.56 | 0.66 | 0.97 | 0.89 | 0.00 | 0.00 | 0.66 | 0.51 | 0.70 | 0.81 |
|  |  |  | palmitoleate (16:1n7) | **3.51** | **3.24** | 1.05 | 0.97 |  |  |  | 0.00 | 0.00 | 0.00 | 0.00 | 0.55 | 0.65 | 0.89 | 0.87 | 0.00 | 0.00 | 0.60 | 0.48 | 0.74 | 0.82 |
|  |  |  | margarate (17:0) | 1.13 | 0.84 | **1.26** | 0.94 |  |  |  | 0.30 | 0.14 | 0.15 | 0.07 | 0.06 | 0.19 | 0.58 | 0.79 | 0.77 | 0.22 | 0.33 | 0.37 | 0.09 | 0.75 |
|  |  |  | 10-heptadecenoate (17:1n7) | **2.07** | **1.51** | 1.20 | 0.88 |  |  |  | 0.00 | 0.00 | 0.01 | 0.01 | 0.13 | 0.29 | 0.60 | 0.80 | 0.00 | 0.00 | 0.46 | 0.43 | 0.15 | 0.75 |
|  |  |  | stearate (18:0) | 1.15 | 1.12 | 1.01 | 0.98 |  |  |  | 0.16 | 0.10 | 0.21 | 0.09 | 0.94 | 0.83 | 0.93 | 0.88 | 0.06 | 0.03 | 0.99 | 0.62 | 0.91 | 0.86 |
|  |  |  | oleate (18:1n9) | **2.08** | **1.78** | 1.22 | 1.04 |  |  |  | 0.00 | 0.00 | 0.00 | 0.00 | 0.13 | 0.30 | 0.56 | 0.78 | 0.00 | 0.00 | 0.14 | 0.24 | 0.50 | 0.80 |
|  |  |  | cis-vaccenate (18:1n7) | **2.17** | **1.93** | 1.06 | 0.94 |  |  |  | 0.00 | 0.00 | 0.00 | 0.00 | 0.69 | 0.73 | 0.84 | 0.87 | 0.00 | 0.00 | 0.89 | 0.60 | 0.68 | 0.81 |
|  |  |  | stearidonate (18:4n3) | **2.40** | **2.75** | 0.77 | 0.89 |  |  |  | 0.00 | 0.00 | 0.00 | 0.00 | 0.19 | 0.37 | 0.46 | 0.75 | 0.00 | 0.00 | 0.15 | 0.25 | 0.67 | 0.81 |
|  |  |  | nonadecanoate (19:0) | 1.01 | 0.80 | 1.17 | 0.93 |  |  |  | 0.93 | 0.28 | 0.10 | 0.06 | 0.20 | 0.38 | 0.64 | 0.82 | 0.27 | 0.09 | 0.55 | 0.46 | 0.22 | 0.75 |
|  |  |  | 10-nonadecenoate (19:1n9) | 1.24 | 0.84 | **1.32** | 0.90 |  |  |  | 0.14 | 0.09 | 0.31 | 0.12 | 0.04 | 0.16 | 0.67 | 0.84 | 0.72 | 0.21 | 0.23 | 0.32 | 0.08 | 0.75 |
|  |  |  | eicosenoate (20:1n9 or 11) | 1.21 | 0.95 | 1.18 | 0.92 |  |  |  | 0.17 | 0.11 | 0.85 | 0.26 | 0.19 | 0.37 | 0.81 | 0.87 | 0.40 | 0.13 | 0.44 | 0.43 | 0.27 | 0.75 |
|  |  |  | dihomo-linoleate (20:2n6) | **1.43** | 1.18 | 1.13 | 0.93 |  |  |  | 0.01 | 0.02 | 0.21 | 0.09 | 0.26 | 0.44 | 0.75 | 0.85 | 0.01 | 0.01 | 0.56 | 0.46 | 0.30 | 0.75 |
|  |  |  | mead acid (20:3n9) | **2.09** | **2.30** | 0.79 | 0.87 |  |  |  | 0.00 | 0.00 | 0.00 | 0.00 | 0.11 | 0.27 | 0.49 | 0.76 | 0.00 | 0.00 | 0.11 | 0.20 | 0.50 | 0.80 |
|  |  |  | arachidonate (20:4n6) | 1.15 | **1.17** | 1.04 | 1.05 |  |  |  | 0.15 | 0.10 | 0.08 | 0.05 | 0.61 | 0.67 | 0.43 | 0.75 | 0.03 | 0.02 | 0.36 | 0.38 | 0.84 | 0.83 |
|  |  |  | docosadienoate (22:2n6) | **1.49** | 1.18 | **1.24** | 0.98 |  |  |  | 0.01 | 0.01 | 0.25 | 0.10 | 0.10 | 0.27 | 0.91 | 0.87 | 0.01 | 0.01 | 0.26 | 0.34 | 0.21 | 0.75 |
|  |  |  | adrenate (22:4n6) | **1.32** | 1.04 | 1.18 | 0.93 |  |  |  | 0.02 | 0.02 | 0.68 | 0.22 | 0.11 | 0.28 | 0.63 | 0.81 | 0.05 | 0.02 | 0.43 | 0.42 | 0.14 | 0.75 |
|  |  | Fatty acid, methyl ester | palmitate, methyl ester | **1.53** | **1.35** | 0.98 | 0.87 |  |  |  | 0.00 | 0.01 | 0.06 | 0.04 | 0.94 | 0.83 | 0.32 | 0.67 | 0.00 | 0.00 | 0.51 | 0.45 | 0.45 | 0.80 |
|  |  | Fatty acid, monohydroxy | 2-hydroxystearate | 1.16 | **1.52** | 0.80 | 1.04 |  |  |  | 0.24 | 0.13 | 0.01 | 0.01 | 0.16 | 0.33 | 0.85 | 0.87 | 0.01 | 0.01 | 0.38 | 0.39 | 0.25 | 0.75 |
|  |  |  | 2-hydroxypalmitate | **1.25** | 1.10 | 0.90 | 0.79 |  |  |  | 0.10 | 0.07 | 0.34 | 0.13 | 0.42 | 0.59 | 0.13 | 0.44 | 0.07 | 0.03 | 0.10 | 0.20 | 0.60 | 0.80 |
|  |  |  | 13-HODE + 9-HODE | 1.15 | 1.12 | 1.07 | 1.05 |  |  |  | 0.26 | 0.13 | 0.43 | 0.16 | 0.55 | 0.65 | 0.80 | 0.87 | 0.18 | 0.07 | 0.55 | 0.46 | 0.81 | 0.83 |
|  |  | Fatty acid, dicarboxylate | 2-hydroxyglutarate | **1.45** | 1.18 | 0.90 | **0.73** |  |  |  | 0.00 | 0.01 | 0.17 | 0.08 | 0.48 | 0.62 | 0.02 | 0.19 | 0.00 | 0.00 | 0.03 | 0.07 | 0.20 | 0.75 |
|  |  |  | tetradecanedioate | 1.10 | **1.37** | **1.41** | **1.75** |  |  |  | 0.55 | 0.21 | 0.02 | 0.02 | 0.02 | 0.10 | 0.00 | 0.03 | 0.04 | 0.02 | 0.00 | 0.00 | 0.19 | 0.75 |
|  |  |  | hexadecanedioate | 1.17 | 1.21 | **1.24** | **1.28** |  |  |  | 0.24 | 0.13 | 0.13 | 0.07 | 0.10 | 0.27 | 0.05 | 0.31 | 0.06 | 0.03 | 0.01 | 0.04 | 0.81 | 0.83 |
|  |  | Fatty acid, branched | 15-methylpalmitate (isobar with 2-methylpalmitate) | **1.86** | **1.55** | 1.11 | 0.92 |  |  |  | 0.00 | 0.00 | 0.00 | 0.00 | 0.31 | 0.50 | 0.60 | 0.80 | 0.00 | 0.00 | 0.72 | 0.53 | 0.27 | 0.75 |
|  |  |  | 17-methylstearate | 0.89 | 0.81 | 1.09 | 1.00 |  |  |  | 0.38 | 0.17 | 0.25 | 0.10 | 0.64 | 0.70 | 0.85 | 0.87 | 0.16 | 0.06 | 0.64 | 0.51 | 0.84 | 0.83 |
|  |  | Eicosanoid | prostaglandin A2 | **0.81** | **1.25** | **0.77** | 1.18 |  |  |  | 0.05 | 0.04 | 0.06 | 0.04 | 0.02 | 0.10 | 0.12 | 0.44 | 0.91 | 0.25 | 0.55 | 0.46 | 0.01 | 0.46 |
|  |  |  | prostaglandin D2 | **0.71** | 1.01 | 1.00 | **1.42** |  |  |  | 0.01 | 0.02 | 0.98 | 0.29 | 0.94 | 0.83 | 0.01 | 0.11 | 0.06 | 0.03 | 0.04 | 0.10 | 0.05 | 0.75 |
|  |  |  | prostaglandin E1 | 1.12 | **1.50** | 0.89 | 1.18 |  |  |  | 0.33 | 0.15 | 0.00 | 0.00 | 0.25 | 0.44 | 0.15 | 0.44 | 0.00 | 0.00 | 0.83 | 0.58 | 0.07 | 0.75 |
|  |  |  | prostaglandin E2 | 0.93 | **1.42** | **0.72** | 1.10 |  |  |  | 0.63 | 0.21 | 0.01 | 0.01 | 0.02 | 0.10 | 0.47 | 0.75 | 0.12 | 0.05 | 0.22 | 0.31 | 0.03 | 0.74 |
|  |  |  | prostaglandin I2 | 1.08 | **1.30** | **0.83** | 1.00 |  |  |  | 0.56 | 0.21 | 0.02 | 0.02 | 0.08 | 0.24 | 0.86 | 0.87 | 0.03 | 0.02 | 0.26 | 0.34 | 0.17 | 0.75 |
|  |  |  | 6-keto prostaglandin F1alpha | 1.08 | **1.27** | **0.85** | 0.99 |  |  |  | 0.51 | 0.20 | 0.02 | 0.02 | 0.09 | 0.26 | 0.91 | 0.87 | 0.03 | 0.02 | 0.26 | 0.34 | 0.20 | 0.75 |
|  |  |  | 12-HEPE | **1.66** | **1.93** | 0.88 | 1.02 |  |  |  | 0.00 | 0.00 | 0.00 | 0.00 | 0.19 | 0.37 | 0.77 | 0.85 | 0.00 | 0.00 | 0.46 | 0.43 | 0.25 | 0.75 |
|  |  | Endocannabinoid | palmitoyl ethanolamide | **1.26** | 1.20 | **0.66** | **0.63** |  |  |  | 0.06 | 0.05 | 0.16 | 0.07 | 0.01 | 0.05 | 0.00 | 0.08 | 0.02 | 0.01 | 0.00 | 0.00 | 0.74 | 0.82 |
|  |  | Fatty acid metabolism (also BCAA metabolism) | propionylcarnitine | **0.64** | 0.93 | **0.73** | 1.06 |  |  |  | 0.00 | 0.00 | 0.54 | 0.19 | 0.01 | 0.05 | 0.52 | 0.76 | 0.00 | 0.00 | 0.14 | 0.24 | 0.02 | 0.67 |
|  |  |  | butyrylcarnitine | **0.74** | 0.87 | **0.84** | 0.99 |  |  |  | 0.01 | 0.02 | 0.32 | 0.12 | 0.09 | 0.26 | 0.90 | 0.87 | 0.02 | 0.01 | 0.20 | 0.28 | 0.26 | 0.75 |
|  |  |  | hydroxybutyrylcarnitine* | **1.82** | 1.15 | **2.08** | 1.31 |  |  |  | 0.01 | 0.02 | 0.99 | 0.29 | 0.00 | 0.02 | 0.46 | 0.75 | 0.06 | 0.03 | 0.00 | 0.02 | 0.05 | 0.75 |
|  |  | Carnitine metabolism | deoxycarnitine | **0.53** | **0.76** | **0.58** | **0.84** |  |  |  | 0.00 | 0.00 | 0.01 | 0.01 | 0.00 | 0.01 | 0.07 | 0.33 | 0.00 | 0.00 | 0.00 | 0.00 | 0.07 | 0.75 |
|  |  |  | carnitine | 0.91 | 1.06 | **0.79** | 0.91 |  |  |  | 0.40 | 0.17 | 0.52 | 0.18 | 0.03 | 0.11 | 0.41 | 0.75 | 0.89 | 0.25 | 0.03 | 0.08 | 0.29 | 0.75 |
|  |  |  | 3-dehydrocarnitine* | **0.79** | 1.01 | **0.80** | 1.02 |  |  |  | 0.03 | 0.03 | 0.87 | 0.26 | 0.03 | 0.14 | 0.78 | 0.85 | 0.13 | 0.05 | 0.17 | 0.26 | 0.09 | 0.75 |
|  |  |  | acetylcarnitine | **0.80** | 0.85 | 0.91 | 0.97 |  |  |  | 0.10 | 0.07 | 0.16 | 0.07 | 0.54 | 0.65 | 0.72 | 0.85 | 0.03 | 0.02 | 0.49 | 0.45 | 0.86 | 0.84 |
|  |  |  | palmitoylcarnitine | 1.39 | 1.18 | **0.63** | **0.53** |  |  |  | 0.38 | 0.17 | 0.60 | 0.20 | 0.06 | 0.18 | 0.03 | 0.25 | 0.32 | 0.11 | 0.00 | 0.02 | 0.80 | 0.83 |
|  |  |  | stearoylcarnitine | 0.87 | 0.83 | **0.50** | **0.48** |  |  |  | 0.74 | 0.24 | 0.75 | 0.24 | 0.01 | 0.04 | 0.01 | 0.11 | 0.65 | 0.19 | 0.00 | 0.00 | 0.99 | 0.86 |
|  |  |  | oleoylcarnitine | **1.71** | 1.32 | 0.78 | **0.60** |  |  |  | 0.07 | 0.05 | 0.44 | 0.16 | 0.26 | 0.44 | 0.03 | 0.26 | 0.07 | 0.03 | 0.02 | 0.06 | 0.43 | 0.79 |
|  |  | Bile acid metabolism | cholate | 0.51 | **9.12** | 0.37 | 6.76 |  |  |  | 0.69 | 0.23 | 0.03 | 0.03 | 0.16 | 0.34 | 0.23 | 0.56 | 0.20 | 0.07 | 0.89 | 0.60 | 0.07 | 0.75 |
|  |  |  | taurocholate | 0.72 | 3.90 | 0.77 | 4.17 |  |  |  | 0.80 | 0.26 | 0.13 | 0.07 | 0.52 | 0.63 | 0.26 | 0.61 | 0.36 | 0.12 | 0.73 | 0.53 | 0.21 | 0.75 |
|  |  |  | taurohyocholate | 0.44 | 0.89 | 1.79 | 3.59 |  |  |  | 0.36 | 0.17 | 0.42 | 0.15 | 0.87 | 0.82 | 0.13 | 0.44 | 0.94 | 0.25 | 0.33 | 0.37 | 0.23 | 0.75 |
|  |  |  | taurochenodeoxycholate | 0.59 | 5.15 | 0.76 | 6.60 |  |  |  | 0.57 | 0.21 | 0.20 | 0.09 | 0.61 | 0.67 | 0.18 | 0.49 | 0.60 | 0.18 | 0.55 | 0.46 | 0.19 | 0.75 |
|  |  |  | taurodeoxycholate | 0.59 | 2.35 | 1.47 | 5.85 |  |  |  | 0.76 | 0.25 | 0.18 | 0.08 | 0.80 | 0.80 | 0.17 | 0.47 | 0.46 | 0.15 | 0.42 | 0.42 | 0.24 | 0.75 |
|  |  |  | tauroursodeoxycholate | 0.46 | 2.92 | 0.62 | 4.00 |  |  |  | 0.54 | 0.21 | 0.28 | 0.11 | 0.44 | 0.60 | 0.35 | 0.71 | 0.73 | 0.21 | 0.90 | 0.60 | 0.23 | 0.75 |
|  |  |  | tauro(alpha + beta)muricholate | 0.48 | 2.38 | 0.73 | 3.62 |  |  |  | 0.54 | 0.21 | 0.28 | 0.11 | 0.47 | 0.62 | 0.33 | 0.69 | 0.74 | 0.21 | 0.86 | 0.59 | 0.24 | 0.75 |
|  |  | Glycerolipid metabolism | choline phosphate | 0.94 | 0.94 | 0.87 | 0.88 |  |  |  | 0.74 | 0.24 | 0.78 | 0.24 | 0.42 | 0.59 | 0.45 | 0.75 | 0.67 | 0.20 | 0.28 | 0.34 | 0.97 | 0.86 |
|  |  |  | ethanolamine | 1.05 | 1.04 | 0.84 | 0.84 |  |  |  | 0.73 | 0.24 | 0.72 | 0.23 | 0.47 | 0.62 | 0.48 | 0.75 | 0.62 | 0.19 | 0.31 | 0.37 | 0.99 | 0.86 |
|  |  |  | phosphoethanolamine | 1.16 | **1.29** | 0.92 | 1.03 |  |  |  | 0.37 | 0.17 | 0.10 | 0.06 | 0.64 | 0.70 | 0.75 | 0.85 | 0.07 | 0.03 | 0.91 | 0.60 | 0.58 | 0.80 |
|  |  |  | glycerol | **1.60** | **1.52** | 1.05 | 0.99 |  |  |  | 0.00 | 0.00 | 0.00 | 0.00 | 0.68 | 0.72 | 0.88 | 0.87 | 0.00 | 0.00 | 0.69 | 0.52 | 0.86 | 0.84 |
|  |  |  | choline | 1.06 | 1.18 | 0.89 | 0.99 |  |  |  | 0.58 | 0.21 | 0.13 | 0.07 | 0.31 | 0.50 | 0.96 | 0.89 | 0.15 | 0.06 | 0.45 | 0.43 | 0.49 | 0.80 |
|  |  |  | glycerol 3-phosphate (G3P) | **1.43** | **1.46** | 0.77 | 0.78 |  |  |  | 0.03 | 0.03 | 0.02 | 0.02 | 0.11 | 0.27 | 0.20 | 0.52 | 0.00 | 0.00 | 0.04 | 0.10 | 0.82 | 0.83 |
|  |  |  | glycerophosphorylcholine (GPC) | **1.39** | **1.45** | **0.74** | **0.76** |  |  |  | 0.01 | 0.01 | 0.00 | 0.00 | 0.01 | 0.06 | 0.02 | 0.24 | 0.00 | 0.00 | 0.00 | 0.01 | 0.83 | 0.83 |
|  |  |  | cytidine 5'-diphosphocholine | **1.28** | **1.22** | 0.94 | 0.90 |  |  |  | 0.01 | 0.02 | 0.06 | 0.04 | 0.76 | 0.78 | 0.29 | 0.64 | 0.00 | 0.00 | 0.33 | 0.37 | 0.60 | 0.80 |
|  |  |  | cytidine-5'-diphosphoethanolamine | **1.22** | **1.28** | 0.90 | 0.95 |  |  |  | 0.05 | 0.05 | 0.02 | 0.02 | 0.40 | 0.58 | 0.67 | 0.84 | 0.00 | 0.00 | 0.37 | 0.39 | 0.76 | 0.83 |
|  |  | Inositol metabolism | myo-inositol | 0.94 | 1.04 | **0.64** | **0.71** |  |  |  | 0.70 | 0.23 | 0.57 | 0.19 | 0.00 | 0.03 | 0.03 | 0.26 | 0.90 | 0.25 | 0.00 | 0.00 | 0.50 | 0.80 |
|  |  |  | chiro-inositol | 0.76 | 1.27 | **0.52** | 0.87 |  |  |  | 0.18 | 0.11 | 0.18 | 0.08 | 0.00 | 0.02 | 0.29 | 0.64 | 1.00 | 0.27 | 0.00 | 0.01 | 0.06 | 0.75 |
|  |  |  | inositol 1-phosphate (I1P) | **1.27** | **1.58** | 0.84 | 1.04 |  |  |  | 0.07 | 0.05 | 0.00 | 0.00 | 0.17 | 0.36 | 0.73 | 0.85 | 0.00 | 0.00 | 0.46 | 0.43 | 0.23 | 0.75 |
|  |  |  | scyllo-inositol | **0.57** | 0.77 | **0.63** | 0.86 |  |  |  | 0.01 | 0.01 | 0.23 | 0.10 | 0.02 | 0.09 | 0.46 | 0.75 | 0.01 | 0.01 | 0.03 | 0.07 | 0.22 | 0.75 |
|  |  | Ketone bodies | 3-hydroxybutyrate (BHBA) | **2.13** | 1.61 | **1.58** | 1.20 |  |  |  | 0.01 | 0.01 | 0.15 | 0.07 | 0.05 | 0.18 | 0.59 | 0.80 | 0.00 | 0.00 | 0.08 | 0.16 | 0.29 | 0.75 |
|  |  | Lysolipid | 1-palmitoylglycerophosphoethanolamine | **1.62** | **1.91** | 0.97 | 1.14 |  |  |  | 0.06 | 0.05 | 0.01 | 0.01 | 0.72 | 0.75 | 0.51 | 0.76 | 0.00 | 0.00 | 0.83 | 0.58 | 0.47 | 0.80 |
|  |  |  | 2-palmitoylglycerophosphoethanolamine* | **1.84** | **2.18** | 0.90 | 1.07 |  |  |  | 0.03 | 0.03 | 0.01 | 0.01 | 0.69 | 0.73 | 0.70 | 0.85 | 0.00 | 0.00 | 1.00 | 0.62 | 0.58 | 0.80 |
|  |  |  | 1-stearoylglycerophosphoethanolamine | 1.75 | **2.36** | 0.87 | 1.17 |  |  |  | 0.31 | 0.15 | 0.06 | 0.04 | 0.59 | 0.66 | 0.69 | 0.85 | 0.04 | 0.02 | 0.92 | 0.60 | 0.51 | 0.80 |
|  |  |  | 1-oleoylglycerophosphoethanolamine | **1.96** | **2.72** | 0.83 | 1.15 |  |  |  | 0.05 | 0.05 | 0.00 | 0.00 | 0.36 | 0.54 | 0.48 | 0.75 | 0.00 | 0.00 | 0.87 | 0.59 | 0.25 | 0.75 |
|  |  |  | 2-oleoylglycerophosphoethanolamine* | **1.97** | **2.15** | 0.92 | 1.01 |  |  |  | 0.03 | 0.03 | 0.01 | 0.01 | 0.80 | 0.80 | 0.88 | 0.87 | 0.00 | 0.00 | 0.94 | 0.61 | 0.77 | 0.83 |
|  |  |  | 1-linoleoylglycerophosphoethanolamine* | 1.36 | **1.72** | 0.82 | 1.04 |  |  |  | 0.26 | 0.13 | 0.05 | 0.04 | 0.39 | 0.56 | 0.97 | 0.89 | 0.03 | 0.02 | 0.56 | 0.46 | 0.52 | 0.80 |
|  |  |  | 2-linoleoylglycerophosphoethanolamine* | 1.13 | 1.52 | 0.81 | 1.09 |  |  |  | 0.98 | 0.29 | 0.22 | 0.09 | 0.45 | 0.61 | 0.61 | 0.80 | 0.39 | 0.13 | 0.86 | 0.59 | 0.37 | 0.77 |
|  |  |  | 1-arachidonoylglycerophosphoethanolamine* | **1.87** | **1.99** | 0.90 | 0.95 |  |  |  | 0.02 | 0.03 | 0.00 | 0.01 | 0.52 | 0.63 | 0.99 | 0.90 | 0.00 | 0.00 | 0.65 | 0.51 | 0.64 | 0.81 |
|  |  |  | 2-arachidonoylglycerophosphoethanolamine* | 0.91 | 2.28 | **0.32** | 0.81 |  |  |  | 0.89 | 0.27 | 0.17 | 0.08 | 0.00 | 0.04 | 0.14 | 0.44 | 0.37 | 0.12 | 0.00 | 0.01 | 0.28 | 0.75 |
|  |  |  | 2-docosapentaenoylglycerophosphoethanolamine* | 1.52 | **4.56** | **0.25** | **0.74** |  |  |  | 0.45 | 0.18 | 0.06 | 0.04 | 0.01 | 0.05 | 0.10 | 0.41 | 0.06 | 0.03 | 0.00 | 0.01 | 0.40 | 0.78 |
|  |  |  | 2-docosahexaenoylglycerophosphoethanolamine* | 1.05 | 2.30 | **0.35** | 0.78 |  |  |  | 0.86 | 0.27 | 0.14 | 0.07 | 0.01 | 0.05 | 0.13 | 0.44 | 0.25 | 0.08 | 0.00 | 0.02 | 0.36 | 0.77 |
|  |  |  | 1-stearoylglycerophosphoglycerol | **1.89** | **1.42** | **1.37** | 1.03 |  |  |  | 0.00 | 0.00 | 0.05 | 0.04 | 0.09 | 0.26 | 0.78 | 0.85 | 0.00 | 0.00 | 0.16 | 0.25 | 0.30 | 0.75 |
|  |  |  | 1-myristoylglycerophosphocholine | 1.29 | **3.10** | **0.22** | **0.53** |  |  |  | 0.43 | 0.18 | 0.04 | 0.03 | 0.00 | 0.02 | 0.02 | 0.25 | 0.04 | 0.02 | 0.00 | 0.00 | 0.34 | 0.76 |
|  |  |  | 2-myristoylglycerophosphocholine* | 1.24 | 1.84 | **0.28** | **0.42** |  |  |  | 0.49 | 0.19 | 0.14 | 0.07 | 0.00 | 0.02 | 0.00 | 0.09 | 0.13 | 0.05 | 0.00 | 0.00 | 0.56 | 0.80 |
|  |  |  | 1-palmitoylglycerophosphocholine | 1.67 | 2.21 | **0.53** | 0.69 |  |  |  | 0.37 | 0.17 | 0.22 | 0.09 | 0.07 | 0.21 | 0.13 | 0.44 | 0.14 | 0.05 | 0.02 | 0.06 | 0.81 | 0.83 |
|  |  |  | 2-palmitoylglycerophosphocholine* | 1.78 | 1.88 | **0.47** | **0.50** |  |  |  | 0.33 | 0.15 | 0.29 | 0.12 | 0.04 | 0.15 | 0.05 | 0.30 | 0.15 | 0.06 | 0.01 | 0.02 | 0.95 | 0.86 |
|  |  |  | 1-palmitoleoylglycerophosphocholine* | 1.63 | **6.94** | **0.23** | 0.97 |  |  |  | 0.24 | 0.13 | 0.01 | 0.01 | 0.01 | 0.04 | 0.25 | 0.60 | 0.01 | 0.01 | 0.01 | 0.02 | 0.21 | 0.75 |
|  |  |  | 2-palmitoleoylglycerophosphocholine* | 1.56 | **2.79** | **0.30** | **0.54** |  |  |  | 0.23 | 0.13 | 0.06 | 0.04 | 0.00 | 0.03 | 0.02 | 0.24 | 0.03 | 0.02 | 0.00 | 0.00 | 0.59 | 0.80 |
|  |  |  | 1-heptadecanoylglycerophosphocholine | **2.88** | 2.57 | 0.93 | 0.83 |  |  |  | 0.10 | 0.07 | 0.69 | 0.22 | 0.99 | 0.84 | 0.19 | 0.51 | 0.14 | 0.06 | 0.34 | 0.37 | 0.36 | 0.77 |
|  |  |  | 1-stearoylglycerophosphocholine | 2.02 | 2.45 | **0.53** | **0.65** |  |  |  | 0.26 | 0.13 | 0.15 | 0.07 | 0.05 | 0.18 | 0.10 | 0.41 | 0.08 | 0.03 | 0.01 | 0.04 | 0.82 | 0.83 |
|  |  |  | 2-stearoylglycerophosphocholine* | **3.70** | 3.06 | 0.66 | **0.54** |  |  |  | 0.02 | 0.02 | 0.34 | 0.13 | 0.57 | 0.66 | 0.05 | 0.30 | 0.02 | 0.01 | 0.07 | 0.14 | 0.30 | 0.75 |
|  |  |  | 1-oleoylglycerophosphocholine | 2.33 | 3.92 | **0.38** | **0.63** |  |  |  | 0.15 | 0.10 | 0.12 | 0.07 | 0.06 | 0.18 | 0.07 | 0.34 | 0.04 | 0.02 | 0.01 | 0.04 | 0.94 | 0.86 |
|  |  |  | 2-oleoylglycerophosphocholine* | 2.31 | 2.20 | 0.64 | **0.61** |  |  |  | 0.11 | 0.07 | 0.35 | 0.13 | 0.17 | 0.36 | 0.04 | 0.30 | 0.08 | 0.03 | 0.02 | 0.06 | 0.61 | 0.80 |
|  |  |  | 1-linoleoylglycerophosphocholine | 1.27 | **3.78** | **0.22** | **0.65** |  |  |  | 0.35 | 0.16 | 0.01 | 0.02 | 0.00 | 0.02 | 0.06 | 0.32 | 0.02 | 0.01 | 0.00 | 0.00 | 0.25 | 0.75 |
|  |  |  | 2-linoleoylglycerophosphocholine* | 0.85 | **3.63** | **0.21** | 0.91 |  |  |  | 0.95 | 0.28 | 0.04 | 0.03 | 0.00 | 0.02 | 0.19 | 0.51 | 0.16 | 0.06 | 0.00 | 0.01 | 0.14 | 0.75 |
|  |  |  | 1-arachidoylglycerophosphocholine | 1.37 | **2.90** | **0.28** | **0.60** |  |  |  | 0.23 | 0.13 | 0.03 | 0.03 | 0.00 | 0.02 | 0.01 | 0.15 | 0.02 | 0.01 | 0.00 | 0.00 | 0.46 | 0.80 |
|  |  |  | 1-arachidonoylglycerophosphocholine* | 1.52 | **4.59** | **0.21** | 0.63 |  |  |  | 0.41 | 0.18 | 0.02 | 0.02 | 0.00 | 0.02 | 0.10 | 0.41 | 0.03 | 0.02 | 0.00 | 0.01 | 0.25 | 0.75 |
|  |  |  | 2-arachidonoylglycerophosphocholine* | 0.98 | **4.12** | **0.20** | 0.85 |  |  |  | 0.86 | 0.27 | 0.08 | 0.05 | 0.00 | 0.03 | 0.15 | 0.44 | 0.17 | 0.06 | 0.00 | 0.01 | 0.25 | 0.75 |
|  |  |  | 1-docosapentaenoylglycerophosphocholine* | **2.50** | **2.45** | **0.55** | **0.54** |  |  |  | 0.01 | 0.02 | 0.04 | 0.03 | 0.03 | 0.14 | 0.01 | 0.12 | 0.00 | 0.00 | 0.00 | 0.01 | 0.66 | 0.81 |
|  |  |  | 2-docosapentaenoylglycerophosphocholine* | 1.92 | **6.55** | **0.21** | 0.73 |  |  |  | 0.11 | 0.07 | 0.00 | 0.00 | 0.00 | 0.02 | 0.11 | 0.42 | 0.00 | 0.00 | 0.00 | 0.01 | 0.24 | 0.75 |
|  |  |  | 1-docosahexaenoylglycerophosphocholine* | 1.11 | 5.12 | **0.23** | 1.07 |  |  |  | 0.76 | 0.25 | 0.11 | 0.06 | 0.03 | 0.14 | 0.76 | 0.85 | 0.35 | 0.11 | 0.08 | 0.16 | 0.18 | 0.75 |
|  |  |  | 2-docosahexaenoylglycerophosphocholine* | 1.21 | **3.59** | **0.26** | 0.77 |  |  |  | 0.59 | 0.21 | 0.10 | 0.06 | 0.01 | 0.05 | 0.11 | 0.43 | 0.12 | 0.05 | 0.00 | 0.02 | 0.41 | 0.78 |
|  |  |  | 1-palmitoylglycerophosphoinositol* | 1.48 | **2.15** | **0.68** | 1.00 |  |  |  | 0.23 | 0.12 | 0.00 | 0.01 | 0.07 | 0.21 | 0.97 | 0.89 | 0.00 | 0.00 | 0.20 | 0.29 | 0.18 | 0.75 |
|  |  |  | 1-stearoylglycerophosphoinositol | 1.75 | **2.41** | 0.74 | 1.02 |  |  |  | 0.20 | 0.12 | 0.00 | 0.01 | 0.12 | 0.28 | 0.89 | 0.87 | 0.00 | 0.00 | 0.31 | 0.37 | 0.23 | 0.75 |
|  |  |  | 1-oleoylglycerophosphoinositol* | **2.07** | **2.38** | 0.97 | 1.11 |  |  |  | 0.01 | 0.02 | 0.00 | 0.00 | 0.87 | 0.82 | 0.29 | 0.64 | 0.00 | 0.00 | 0.52 | 0.46 | 0.39 | 0.78 |
|  |  |  | 1-arachidonoylglycerophosphoinositol* | 1.45 | **1.68** | 0.75 | 0.87 |  |  |  | 0.26 | 0.13 | 0.02 | 0.02 | 0.12 | 0.28 | 0.76 | 0.85 | 0.02 | 0.01 | 0.19 | 0.27 | 0.37 | 0.77 |
|  |  |  | 2-arachidonoylglycerophosphoinositol* | 1.45 | **2.50** | **0.57** | 0.99 |  |  |  | 0.59 | 0.21 | 0.01 | 0.01 | 0.05 | 0.18 | 0.73 | 0.85 | 0.02 | 0.01 | 0.24 | 0.33 | 0.10 | 0.75 |
|  |  |  | 1-palmitoylplasmenylethanolamine* | 1.60 | **2.60** | 0.71 | 1.15 |  |  |  | 0.20 | 0.12 | 0.00 | 0.00 | 0.13 | 0.29 | 0.55 | 0.78 | 0.00 | 0.00 | 0.50 | 0.45 | 0.14 | 0.75 |
|  |  | Monoacylglycerol | 2-palmitoylglycerol (2-monopalmitin) | **1.44** | 1.08 | 1.08 | 0.81 |  |  |  | 0.03 | 0.03 | 0.54 | 0.19 | 0.57 | 0.66 | 0.31 | 0.66 | 0.05 | 0.03 | 0.75 | 0.54 | 0.27 | 0.75 |
|  |  |  | 1-stearoylglycerol (1-monostearin) | **1.31** | 1.05 | 0.97 | **0.78** |  |  |  | 0.05 | 0.05 | 0.64 | 0.21 | 0.79 | 0.80 | 0.08 | 0.37 | 0.09 | 0.04 | 0.15 | 0.25 | 0.28 | 0.75 |
|  |  |  | 1-oleoylglycerol (1-monoolein) | **1.99** | 1.21 | **1.46** | 0.88 |  |  |  | 0.01 | 0.01 | 0.64 | 0.21 | 0.09 | 0.26 | 0.45 | 0.75 | 0.02 | 0.01 | 0.50 | 0.45 | 0.09 | 0.75 |
|  |  |  | 2-oleoylglycerol (2-monoolein) | 1.41 | **0.76** | **1.47** | 0.79 |  |  |  | 0.18 | 0.11 | 0.09 | 0.05 | 0.10 | 0.27 | 0.16 | 0.47 | 0.78 | 0.22 | 0.85 | 0.59 | 0.03 | 0.74 |
|  |  |  | 1-linoleoylglycerol (1-monolinolein) | 1.39 | **0.61** | **1.84** | 0.81 |  |  |  | 0.16 | 0.10 | 0.04 | 0.03 | 0.01 | 0.05 | 0.43 | 0.75 | 0.59 | 0.18 | 0.15 | 0.25 | 0.01 | 0.52 |
|  |  |  | 2-linoleoylglycerol (2-monolinolein) | 1.11 | **0.59** | **1.72** | 0.92 |  |  |  | 0.59 | 0.21 | 0.02 | 0.02 | 0.01 | 0.05 | 0.86 | 0.87 | 0.17 | 0.06 | 0.07 | 0.14 | 0.04 | 0.75 |
|  |  | Diacylglycerol | 1,2-dipalmitoylglycerol | 1.33 | 0.90 | 1.08 | 0.73 |  |  |  | 0.20 | 0.12 | 0.89 | 0.27 | 0.98 | 0.84 | 0.17 | 0.47 | 0.42 | 0.13 | 0.33 | 0.37 | 0.31 | 0.75 |
|  |  |  | 1,3-dipalmitoylglycerol | 1.26 | 0.85 | 1.10 | 0.74 |  |  |  | 0.23 | 0.13 | 0.49 | 0.17 | 0.53 | 0.64 | 0.21 | 0.54 | 0.71 | 0.21 | 0.66 | 0.51 | 0.19 | 0.75 |
|  |  | Sphingolipid | sphinganine | 1.43 | 1.92 | 0.84 | 1.13 |  |  |  | 0.44 | 0.18 | 0.18 | 0.08 | 0.19 | 0.37 | 0.47 | 0.75 | 0.14 | 0.05 | 0.16 | 0.25 | 0.67 | 0.81 |
|  |  |  | sphingosine | 1.47 | 1.88 | **0.56** | 0.72 |  |  |  | 0.41 | 0.17 | 0.22 | 0.09 | 0.04 | 0.16 | 0.10 | 0.41 | 0.15 | 0.06 | 0.01 | 0.04 | 0.77 | 0.83 |
|  |  |  | palmitoyl sphingomyelin | 1.19 | 1.10 | 0.98 | 0.91 |  |  |  | 0.21 | 0.12 | 0.55 | 0.19 | 0.95 | 0.83 | 0.46 | 0.75 | 0.19 | 0.07 | 0.57 | 0.47 | 0.63 | 0.81 |
|  |  |  | stearoyl sphingomyelin | 0.87 | 0.80 | 0.97 | 0.89 |  |  |  | 0.48 | 0.19 | 0.24 | 0.10 | 0.85 | 0.82 | 0.50 | 0.76 | 0.19 | 0.07 | 0.54 | 0.46 | 0.73 | 0.82 |
|  |  | Sterol/Steroid | lathosterol | 0.91 | 1.04 | 0.91 | 1.04 |  |  |  | 0.98 | 0.29 | 0.90 | 0.27 | 0.94 | 0.83 | 0.86 | 0.87 | 0.91 | 0.25 | 0.85 | 0.59 | 0.94 | 0.86 |
|  |  |  | squalene | 1.70 | 0.92 | 1.35 | 0.73 |  |  |  | 0.51 | 0.20 | 0.95 | 0.28 | 0.39 | 0.57 | 0.89 | 0.87 | 0.68 | 0.20 | 0.48 | 0.44 | 0.61 | 0.80 |
|  |  |  | cholesterol | 1.08 | 0.93 | 1.00 | 0.87 |  |  |  | 0.42 | 0.18 | 0.53 | 0.19 | 0.99 | 0.84 | 0.16 | 0.47 | 0.89 | 0.25 | 0.32 | 0.37 | 0.31 | 0.75 |
|  |  |  | dihydrocholesterol | **2.03** | **1.60** | 1.02 | 0.80 |  |  |  | 0.00 | 0.01 | 0.10 | 0.06 | 0.99 | 0.84 | 0.14 | 0.44 | 0.00 | 0.00 | 0.30 | 0.36 | 0.29 | 0.75 |
|  |  |  | 7-alpha-hydroxycholesterol | **1.67** | 1.23 | 0.93 | **0.69** |  |  |  | 0.02 | 0.03 | 0.41 | 0.15 | 0.83 | 0.82 | 0.09 | 0.40 | 0.03 | 0.02 | 0.17 | 0.26 | 0.29 | 0.75 |
|  |  |  | 7-beta-hydroxycholesterol | **1.68** | 1.32 | 0.84 | **0.66** |  |  |  | 0.01 | 0.02 | 0.26 | 0.11 | 0.56 | 0.66 | 0.04 | 0.29 | 0.01 | 0.01 | 0.06 | 0.13 | 0.27 | 0.75 |
|  |  |  | 7-ketocholesterol | **2.56** | 1.29 | 1.05 | **0.53** |  |  |  | 0.00 | 0.00 | 0.28 | 0.11 | 0.87 | 0.82 | 0.03 | 0.25 | 0.00 | 0.00 | 0.13 | 0.24 | 0.09 | 0.75 |
|  |  |  | corticosterone | 0.88 | 1.12 | 1.20 | **1.53** |  |  |  | 0.46 | 0.19 | 0.46 | 0.16 | 0.21 | 0.39 | 0.01 | 0.14 | 1.00 | 0.27 | 0.01 | 0.03 | 0.30 | 0.75 |
|  |  |  | beta-sitosterol | 1.32 | 0.98 | 1.06 | 0.78 |  |  |  | 0.16 | 0.10 | 1.00 | 0.29 | 0.91 | 0.83 | 0.20 | 0.52 | 0.32 | 0.11 | 0.40 | 0.41 | 0.32 | 0.76 |
|  |  |  | campesterol | 1.23 | 1.01 | 1.07 | 0.87 |  |  |  | 0.19 | 0.11 | 0.90 | 0.27 | 0.87 | 0.82 | 0.30 | 0.65 | 0.31 | 0.11 | 0.54 | 0.46 | 0.40 | 0.78 |
|  | Nucleotide | Purine metabolism, (hypo)xanthine/inosine containing | xanthine | 1.04 | 1.22 | 0.99 | 1.15 |  |  |  | 0.59 | 0.21 | 0.15 | 0.07 | 0.98 | 0.84 | 0.37 | 0.71 | 0.16 | 0.06 | 0.53 | 0.46 | 0.51 | 0.80 |
|  |  |  | xanthosine | **1.42** | **1.49** | 1.04 | 1.10 |  |  |  | 0.02 | 0.02 | 0.02 | 0.02 | 0.58 | 0.66 | 0.54 | 0.78 | 0.00 | 0.00 | 0.41 | 0.42 | 0.97 | 0.86 |
|  |  |  | hypoxanthine | 0.93 | 1.02 | 0.97 | 1.07 |  |  |  | 0.56 | 0.21 | 0.75 | 0.23 | 0.80 | 0.80 | 0.52 | 0.76 | 0.86 | 0.24 | 0.78 | 0.56 | 0.52 | 0.80 |
|  |  |  | inosine | 0.94 | 1.08 | 0.94 | 1.07 |  |  |  | 0.73 | 0.24 | 0.41 | 0.15 | 0.67 | 0.72 | 0.46 | 0.75 | 0.74 | 0.21 | 0.82 | 0.58 | 0.41 | 0.78 |
|  |  |  | inosine 5'-monophosphate (IMP) | **2.12** | **2.67** | 0.75 | 0.95 |  |  |  | 0.01 | 0.01 | 0.01 | 0.01 | 0.47 | 0.62 | 0.47 | 0.75 | 0.00 | 0.00 | 0.31 | 0.37 | 0.99 | 0.86 |
|  |  | Purine metabolism, adenine containing | adenine | **1.22** | **1.32** | 1.00 | 1.08 |  |  |  | 0.08 | 0.06 | 0.02 | 0.02 | 0.90 | 0.83 | 0.53 | 0.77 | 0.00 | 0.00 | 0.72 | 0.53 | 0.60 | 0.80 |
|  |  |  | adenosine | 0.86 | **1.31** | **0.67** | 1.03 |  |  |  | 0.56 | 0.21 | 0.09 | 0.05 | 0.04 | 0.16 | 0.83 | 0.87 | 0.42 | 0.13 | 0.19 | 0.27 | 0.11 | 0.75 |
|  |  |  | N1-methyladenosine | 1.09 | 1.14 | **0.76** | **0.79** |  |  |  | 0.43 | 0.18 | 0.36 | 0.14 | 0.06 | 0.20 | 0.08 | 0.37 | 0.23 | 0.08 | 0.01 | 0.04 | 0.93 | 0.86 |
|  |  |  | adenosine 2'-monophosphate (2'-AMP) | **1.60** | **1.42** | 0.93 | 0.83 |  |  |  | 0.03 | 0.03 | 0.07 | 0.05 | 0.65 | 0.71 | 0.36 | 0.71 | 0.01 | 0.01 | 0.34 | 0.37 | 0.74 | 0.82 |
|  |  |  | adenosine 3'-monophosphate (3'-AMP) | **1.77** | **2.41** | **0.48** | **0.65** |  |  |  | 0.02 | 0.03 | 0.01 | 0.01 | 0.01 | 0.04 | 0.01 | 0.15 | 0.00 | 0.00 | 0.00 | 0.00 | 0.82 | 0.83 |
|  |  |  | adenosine 5'-monophosphate (AMP) | **1.97** | **1.96** | 0.70 | **0.70** |  |  |  | 0.01 | 0.02 | 0.03 | 0.03 | 0.15 | 0.32 | 0.06 | 0.32 | 0.00 | 0.00 | 0.02 | 0.06 | 0.74 | 0.82 |
|  |  |  | adenosine 3',5'-cyclic monophosphate (cAMP) | 1.11 | 1.18 | 0.88 | 0.94 |  |  |  | 0.39 | 0.17 | 0.12 | 0.07 | 0.30 | 0.50 | 0.75 | 0.85 | 0.09 | 0.04 | 0.34 | 0.37 | 0.61 | 0.80 |
|  |  |  | adenylosuccinate | **1.78** | **2.01** | 0.75 | 0.85 |  |  |  | 0.06 | 0.05 | 0.09 | 0.05 | 0.48 | 0.62 | 0.39 | 0.73 | 0.01 | 0.01 | 0.27 | 0.34 | 0.91 | 0.86 |
|  |  | Purine metabolism, guanine containing | guanosine | **0.67** | 0.84 | 0.95 | 1.18 |  |  |  | 0.03 | 0.03 | 0.43 | 0.16 | 0.73 | 0.76 | 0.27 | 0.64 | 0.04 | 0.02 | 0.59 | 0.47 | 0.31 | 0.75 |
|  |  |  | guanosine 5'- monophosphate (5'-GMP) | 1.45 | 1.50 | 0.67 | **0.69** |  |  |  | 0.21 | 0.12 | 0.47 | 0.17 | 0.15 | 0.32 | 0.05 | 0.31 | 0.16 | 0.06 | 0.02 | 0.06 | 0.69 | 0.81 |
|  |  |  | guanosine 5'-diphospho-fucose | 1.05 | **1.32** | **0.77** | 0.97 |  |  |  | 0.50 | 0.20 | 0.01 | 0.01 | 0.02 | 0.10 | 0.77 | 0.85 | 0.02 | 0.01 | 0.06 | 0.14 | 0.14 | 0.75 |
|  |  |  | guanosine 3'-monophosphate (3'-GMP) | **1.75** | **1.95** | **0.51** | **0.56** |  |  |  | 0.06 | 0.05 | 0.07 | 0.04 | 0.01 | 0.05 | 0.01 | 0.11 | 0.01 | 0.01 | 0.00 | 0.00 | 0.95 | 0.86 |
|  |  | Purine metabolism, urate metabolism | urate | 1.13 | 1.28 | 1.19 | **1.34** |  |  |  | 0.39 | 0.17 | 0.11 | 0.06 | 0.19 | 0.37 | 0.04 | 0.30 | 0.09 | 0.04 | 0.02 | 0.06 | 0.59 | 0.80 |
|  |  |  | allantoin | 1.01 | **1.56** | 0.60 | 0.93 |  |  |  | 0.60 | 0.21 | 0.06 | 0.04 | 0.15 | 0.32 | 0.96 | 0.89 | 0.09 | 0.04 | 0.28 | 0.35 | 0.31 | 0.75 |
|  |  | Pyrimidine metabolism, cytidine containing | cytidine | 1.08 | **1.31** | 1.10 | **1.33** |  |  |  | 0.51 | 0.20 | 0.05 | 0.04 | 0.48 | 0.62 | 0.04 | 0.30 | 0.06 | 0.03 | 0.06 | 0.12 | 0.33 | 0.76 |
|  |  |  | cytidine 5'-monophosphate (5'-CMP) | 1.02 | 1.04 | 1.06 | 1.09 |  |  |  | 0.81 | 0.26 | 0.72 | 0.23 | 0.36 | 0.54 | 0.30 | 0.65 | 0.67 | 0.20 | 0.17 | 0.26 | 0.94 | 0.86 |
|  |  |  | cytidine-3'-monophosphate (3'-CMP) | 1.05 | **1.98** | **0.34** | **0.65** |  |  |  | 0.61 | 0.21 | 0.09 | 0.05 | 0.00 | 0.02 | 0.03 | 0.25 | 0.12 | 0.05 | 0.00 | 0.00 | 0.39 | 0.78 |
|  |  | Pyrimidine metabolism, orotate containing | orotate | 1.07 | 1.22 | **1.86** | **2.11** |  |  |  | 0.69 | 0.23 | 0.20 | 0.09 | 0.01 | 0.04 | 0.00 | 0.04 | 0.23 | 0.08 | 0.00 | 0.00 | 0.53 | 0.80 |
|  |  | Pyrimidine metabolism, uracil containing | uracil | 1.05 | 1.06 | 1.15 | 1.17 |  |  |  | 0.65 | 0.22 | 0.58 | 0.19 | 0.34 | 0.53 | 0.29 | 0.64 | 0.48 | 0.15 | 0.16 | 0.25 | 0.94 | 0.86 |
|  |  |  | uridine | 1.12 | 1.08 | **1.25** | **1.20** |  |  |  | 0.21 | 0.12 | 0.44 | 0.16 | 0.02 | 0.09 | 0.05 | 0.32 | 0.16 | 0.06 | 0.00 | 0.02 | 0.72 | 0.82 |
|  |  |  | pseudouridine | 1.05 | 1.20 | 0.88 | 1.01 |  |  |  | 0.58 | 0.21 | 0.16 | 0.07 | 0.45 | 0.61 | 0.90 | 0.87 | 0.16 | 0.06 | 0.66 | 0.51 | 0.54 | 0.80 |
|  |  |  | uridine monophosphate (5' or 3') | 1.24 | 1.24 | 0.96 | 0.97 |  |  |  | 0.29 | 0.14 | 0.65 | 0.21 | 0.80 | 0.80 | 0.39 | 0.73 | 0.29 | 0.10 | 0.43 | 0.42 | 0.66 | 0.81 |
|  |  | Purine and pyrimidine metabolism | methylphosphate | **1.18** | 1.18 | 1.05 | 1.05 |  |  |  | 0.09 | 0.06 | 0.10 | 0.06 | 0.58 | 0.66 | 0.63 | 0.81 | 0.02 | 0.01 | 0.47 | 0.43 | 0.96 | 0.86 |
|  | Cofactors and vitamins | Ascorbate and aldarate metabolism | gulono-1,4-lactone | **2.58** | **1.90** | **1.36** | 1.00 |  |  |  | 0.00 | 0.00 | 0.00 | 0.00 | 0.05 | 0.18 | 0.97 | 0.89 | 0.00 | 0.00 | 0.16 | 0.25 | 0.17 | 0.75 |
|  |  |  | ascorbate (Vitamin C) | 1.16 | **1.27** | 0.93 | 1.03 |  |  |  | 0.29 | 0.14 | 0.04 | 0.03 | 0.44 | 0.60 | 0.75 | 0.85 | 0.03 | 0.02 | 0.75 | 0.54 | 0.45 | 0.80 |
|  |  |  | dehydroascorbate | 1.06 | **1.71** | **0.53** | 0.85 |  |  |  | 0.42 | 0.18 | 0.02 | 0.02 | 0.02 | 0.10 | 0.40 | 0.74 | 0.03 | 0.02 | 0.03 | 0.07 | 0.28 | 0.75 |
|  |  |  | threonate | 0.89 | **2.07** | **0.39** | 0.91 |  |  |  | 0.35 | 0.16 | 0.01 | 0.02 | 0.05 | 0.18 | 0.76 | 0.85 | 0.02 | 0.01 | 0.11 | 0.21 | 0.24 | 0.75 |
|  |  |  | glucarate (saccharate) | **0.36** | **0.40** | 0.78 | 0.85 |  |  |  | 0.00 | 0.00 | 0.00 | 0.00 | 0.22 | 0.40 | 0.25 | 0.60 | 0.00 | 0.00 | 0.10 | 0.19 | 0.96 | 0.86 |
|  |  | Folate metabolism | 5-methyltetrahydrofolate (5MeTHF) | 1.03 | **1.23** | **0.71** | **0.85** |  |  |  | 0.61 | 0.21 | 0.04 | 0.03 | 0.00 | 0.02 | 0.10 | 0.41 | 0.07 | 0.03 | 0.00 | 0.01 | 0.25 | 0.75 |
|  |  | Tetrahydrobiopterin metabolism | biopterin | 1.15 | **1.33** | 1.05 | 1.22 |  |  |  | 0.23 | 0.12 | 0.07 | 0.05 | 0.58 | 0.66 | 0.25 | 0.60 | 0.04 | 0.02 | 0.23 | 0.32 | 0.67 | 0.81 |
|  |  | Hemoglobin and porphyrin metabolism | heme | 0.83 | 1.94 | **0.23** | **0.53** |  |  |  | 0.40 | 0.17 | 0.11 | 0.06 | 0.00 | 0.04 | 0.03 | 0.26 | 0.09 | 0.04 | 0.00 | 0.01 | 0.58 | 0.80 |
|  |  | Nicotinate and nicotinamide metabolism | nicotinamide | 1.15 | 1.13 | 0.91 | 0.90 |  |  |  | 0.21 | 0.12 | 0.19 | 0.09 | 0.39 | 0.56 | 0.43 | 0.75 | 0.07 | 0.03 | 0.24 | 0.33 | 0.96 | 0.86 |
|  |  |  | nicotinamide adenine dinucleotide (NAD+) | 0.96 | 1.23 | 0.81 | 1.04 |  |  |  | 0.86 | 0.27 | 0.28 | 0.11 | 0.35 | 0.54 | 0.98 | 0.90 | 0.37 | 0.12 | 0.50 | 0.45 | 0.52 | 0.80 |
|  |  |  | adenosine 5'diphosphoribose | 0.92 | 0.96 | 0.90 | 0.94 |  |  |  | 0.60 | 0.21 | 0.57 | 0.19 | 0.44 | 0.60 | 0.41 | 0.75 | 0.44 | 0.14 | 0.26 | 0.34 | 0.97 | 0.86 |
|  |  |  | trigonelline (N'-methylnicotinate) | 1.08 | **1.55** | **0.57** | **0.82** |  |  |  | 0.37 | 0.17 | 0.00 | 0.00 | 0.00 | 0.00 | 0.06 | 0.32 | 0.00 | 0.00 | 0.00 | 0.00 | 0.03 | 0.74 |
|  |  | Pantothenate and CoA metabolism | pantothenate | **1.26** | **1.40** | 1.01 | 1.12 |  |  |  | 0.03 | 0.03 | 0.00 | 0.01 | 0.86 | 0.82 | 0.30 | 0.65 | 0.00 | 0.00 | 0.39 | 0.41 | 0.54 | 0.80 |
|  |  |  | phosphopantetheine | 0.82 | 0.82 | **1.45** | 1.44 |  |  |  | 0.27 | 0.14 | 0.12 | 0.06 | 0.05 | 0.18 | 0.14 | 0.44 | 0.06 | 0.03 | 0.02 | 0.06 | 0.73 | 0.82 |
|  |  |  | coenzyme A | 0.87 | **0.72** | 1.03 | 0.85 |  |  |  | 0.31 | 0.15 | 0.01 | 0.01 | 0.83 | 0.82 | 0.13 | 0.44 | 0.01 | 0.01 | 0.36 | 0.38 | 0.22 | 0.75 |
|  |  |  | 3'-dephosphocoenzyme A | **1.28** | 1.01 | 1.06 | 0.83 |  |  |  | 0.07 | 0.06 | 0.94 | 0.28 | 0.66 | 0.71 | 0.14 | 0.44 | 0.21 | 0.08 | 0.46 | 0.43 | 0.18 | 0.75 |
|  |  |  | acetyl CoA | 0.82 | **0.75** | 0.84 | **0.78** |  |  |  | 0.14 | 0.10 | 0.06 | 0.04 | 0.23 | 0.41 | 0.10 | 0.41 | 0.02 | 0.01 | 0.05 | 0.11 | 0.73 | 0.82 |
|  |  | Riboflavin metabolism | flavin adenine dinucleotide (FAD) | 1.14 | **1.18** | 0.90 | 0.94 |  |  |  | 0.18 | 0.11 | 0.07 | 0.04 | 0.29 | 0.48 | 0.59 | 0.80 | 0.03 | 0.02 | 0.26 | 0.34 | 0.71 | 0.82 |
|  |  |  | riboflavin (Vitamin B2) | 1.03 | 1.20 | 0.84 | 0.98 |  |  |  | 0.71 | 0.23 | 0.16 | 0.07 | 0.22 | 0.40 | 0.86 | 0.87 | 0.20 | 0.07 | 0.32 | 0.37 | 0.45 | 0.80 |
|  |  |  | flavin mononucleotide (FMN) | **0.43** | 0.69 | 1.40 | **2.22** |  |  |  | 0.01 | 0.01 | 0.26 | 0.11 | 0.16 | 0.33 | 0.00 | 0.08 | 0.01 | 0.01 | 0.00 | 0.01 | 0.23 | 0.75 |
|  |  | Tocopherol metabolism | alpha-tocopherol | **0.64** | **0.71** | 0.93 | 1.01 |  |  |  | 0.01 | 0.02 | 0.02 | 0.02 | 0.55 | 0.65 | 0.70 | 0.85 | 0.00 | 0.00 | 0.49 | 0.44 | 0.88 | 0.85 |
|  | Xenobiotics | Benzoate metabolism | hippurate | **1.66** | **2.76** | **0.61** | 1.02 |  |  |  | 0.01 | 0.02 | 0.00 | 0.00 | 0.03 | 0.14 | 0.99 | 0.90 | 0.00 | 0.00 | 0.13 | 0.23 | 0.13 | 0.75 |
|  |  |  | catechol sulfate | 0.53 | 1.53 | 0.48 | 1.38 |  |  |  | 0.50 | 0.20 | 0.33 | 0.13 | 0.21 | 0.38 | 0.70 | 0.85 | 0.83 | 0.23 | 0.53 | 0.46 | 0.25 | 0.75 |
|  |  |  | benzoate | 1.01 | 0.97 | **1.71** | **1.64** |  |  |  | 0.88 | 0.27 | 0.93 | 0.28 | 0.00 | 0.02 | 0.00 | 0.05 | 0.86 | 0.24 | 0.00 | 0.00 | 0.97 | 0.86 |
|  |  | Chemical | glycerol 2-phosphate | 1.16 | **1.36** | 0.74 | 0.87 |  |  |  | 0.23 | 0.13 | 0.04 | 0.03 | 0.11 | 0.28 | 0.46 | 0.75 | 0.03 | 0.02 | 0.10 | 0.20 | 0.54 | 0.80 |
|  |  | Drug | pentobarbital | 0.70 | 0.84 | 0.86 | 1.02 |  |  |  | 0.34 | 0.16 | 0.46 | 0.16 | 0.85 | 0.82 | 0.98 | 0.90 | 0.24 | 0.08 | 0.90 | 0.60 | 0.88 | 0.85 |
|  |  | Food component/Plant | ergothioneine | 1.05 | **1.31** | **0.59** | **0.73** |  |  |  | 0.64 | 0.22 | 0.05 | 0.04 | 0.00 | 0.02 | 0.04 | 0.30 | 0.09 | 0.04 | 0.00 | 0.00 | 0.28 | 0.75 |
|  |  |  | N-glycolylneuraminate | 1.12 | 0.78 | 1.37 | 0.96 |  |  |  | 0.55 | 0.21 | 0.20 | 0.09 | 0.22 | 0.40 | 0.51 | 0.76 | 0.62 | 0.19 | 0.68 | 0.52 | 0.18 | 0.75 |
|  |  |  | stachydrine | 1.19 | **1.60** | **0.41** | **0.56** |  |  |  | 0.30 | 0.14 | 0.03 | 0.03 | 0.00 | 0.02 | 0.01 | 0.15 | 0.03 | 0.02 | 0.00 | 0.00 | 0.40 | 0.78 |
|  |  |  | equol sulfate | 0.59 | **4.62** | **0.41** | **3.19** |  |  |  | 0.18 | 0.11 | 0.00 | 0.00 | 0.01 | 0.08 | 0.00 | 0.08 | 0.04 | 0.02 | 0.65 | 0.51 | 0.00 | 0.08 |
|  |  | Sugar, sugar substitute, starch | erythritol | **1.59** | **2.01** | **0.60** | **0.76** |  |  |  | 0.01 | 0.01 | 0.00 | 0.00 | 0.02 | 0.08 | 0.06 | 0.33 | 0.00 | 0.00 | 0.00 | 0.02 | 0.65 | 0.81 |
|  |  |  |  |  |  |  |  |  |  |  |  |  |  |  |  |  |  |  |  |  |  |  |  |  |
|  |  |  |  |  |  |  |  |  |  |  |  |  |  |  |  |  |  |  |  |  |  |  |  |  |
