# Supplementary material for: Effect of acute ozone exposure on the lung metabolomes of obese and lean mice
Source: PLoS One. 2017 Jul 13;12(7):e0181017. doi: 10.1371/journal.pone.0181017 (PMC5509247; doi:10.1371/journal.pone.0181017)
Supplement: S1 Method — (DOCX) [file pone.0181017.s002.docx]

**Online supplement**

Effect of the acute ozone exposure on the lung metabolomes of obese and lean mice

Joel A. Mathews^1^, David I. Kasahara^1^, Youngji Cho^1^, Nicki Bell^2^, P. Ross Gunst^2^, Edward Karoly^2^, and Stephanie A. Shore^1^

^1^ Department of Environmental Health, Harvard T.H. Chan School of Public Health, Boston, MA; and ^2^Metabolon Inc., Durham, NC.

**Supplemental Methods**

**Liquid chromatography/Mass Spectrometry (LC/MS, LC/MS^2^)***.*  The LC/MS platform comprised a Waters ACQUITY UPLC and a Thermo-Finnigan LTQ mass spectrometer. The latter consisted of an electrospray ionization (ESI) source and linear ion-trap (LIT) mass analyzer.  Each sample extract was split into two aliquots, dried, and reconstituted in acidic or basic LC-compatible solvents, which contained injection standards at fixed concentrations. One aliquot was analyzed using acidic positive ion optimized conditions and the other using basic negative ion optimized conditions in two independent injections using separate dedicated columns. For extracts reconstituted in acidic conditions, the extracts were gradient eluted using water and methanol both containing 0.1% formic acid. Elution of the basic extracts, also used water/methanol, but contained 6.5mM ammonium bicarbonate. The MS analysis alternated between MS and data-dependent MS^2^ scans using dynamic exclusion.

**Gas chromatography/Mass Spectrometry (GC/MS).** Samples were re-dried under vacuum desiccation for at least 24 hours prior to being derivatized under dry nitrogen using bistrimethyl-silyl-trifluoroacetamide.  The GC column was 5% phenyl and the temperature ramp was from 40° to 300° C over a 16 minute period.  A Thermo-Finnigan Trace DSQ fast-scanning single-quadrupole mass spectrometer using electron impact ionization that was tuned and calibrated for mass resolution and mass accuracy daily was used to analyze the samples.

**Accurate Mass Determination and MS/MS fragmentation (LC/MS), (LC/MS/MS).** The LC/MS portion of the platform used a Waters ACQUITY UPLC and a Thermo-Finnigan LTQ-FT mass spectrometer. The latter had a linear ion-trap (LIT) front end and a Fourier transform ion cyclotron resonance (FT-ICR) mass spectrometer backend.    For ions with counts greater than 2 million, an accurate mass measurement could be performed on the parent ion as well as fragments.  The typical mass error was less than 5 ppm.  For ions with less than two million counts, fragmentation spectra (MS/MS) were typically generated in data dependent manner. Targted MS/MS was employed in the case of lower level signal.
