# Supplementary material for: Effect of acute ozone exposure on the lung metabolomes of obese and lean mice
Source: PLoS One. 2017 Jul 13;12(7):e0181017. doi: 10.1371/journal.pone.0181017 (PMC5509247; doi:10.1371/journal.pone.0181017)
Supplement: S1 Fig — (DOCX) [file pone.0181017.s003.docx]

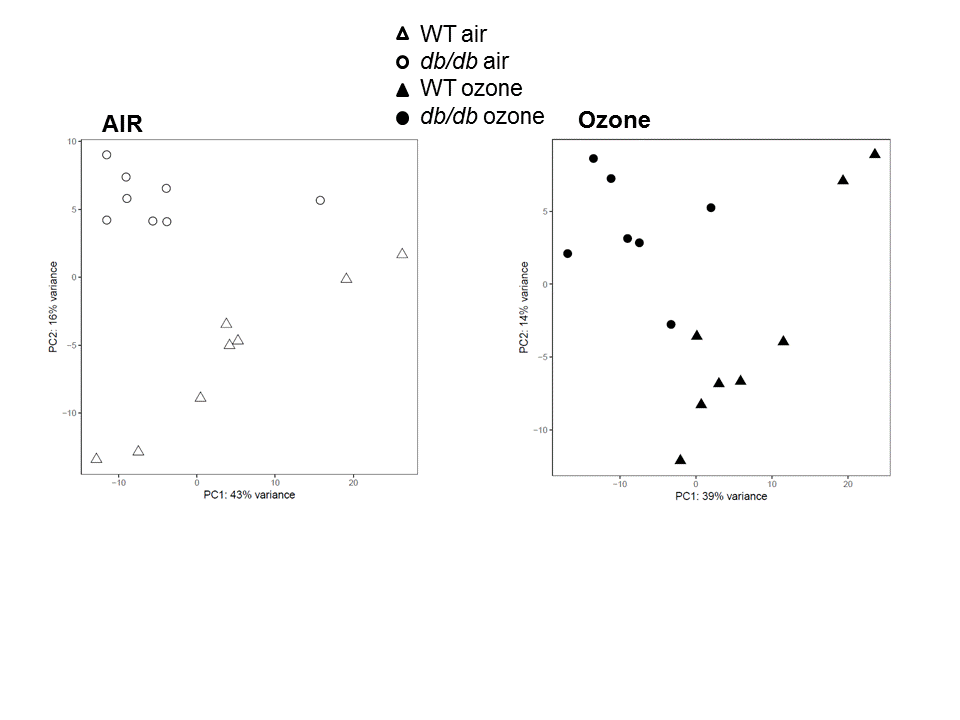


**S1 Figure:** Principal component analysis of lung metabolites from lean wildtype (WT) and obese *db/db* mice exposed to air (left) or ozone (right).
